# Supplementary material for: DoOR 2.0 - Comprehensive Mapping of Drosophila melanogaster Odorant Responses
Source: Sci Rep. 2016 Feb 25;6:21841. doi: 10.1038/srep21841 (PMC4766438; doi:10.1038/srep21841)
Supplement: Supplementary Information [file srep21841-s1.pdf]

DoOR 2.0 - Comprehensive Mapping of *Drosophila melanogaster* Odorant Responses

Daniel Münch[1,*] **and C. Giovanni Galizia**[1]

[1]Neurobiology, University of Konstanz, 78457 Konstanz, Germany
[*]daniel.muench@uni-konstanz.de

ABSTRACT

Odors elicit complex patterns of activated olfactory sensory neurons. Knowing the complete olfactome, i.e. responses in all sensory neurons for all odorants, is desirable to understand olfactory coding. The DoOR project combines all available *Drosophila* odorant response data into a single consensus response matrix. Since its first release many studies were published: receptors were deorphanized and several response profiles were expanded. In this study, we add to the odor-response profiles for four odorant receptors (Or10a, Or42b, Or47b, Or56a). We deorphanize Or69a, showing a broad response spectrum with the best ligands including 3-hydroxyhexanoate, alpha-terpineol, 3-octanol and linalool. We include these datasets into DoOR, and provide a comprehensive update of both code and data. The DoOR project has a web interface for quick queries (http://neuro.uni.kn/DoOR), and a downloadable, open source toolbox written in R, including all processed and original datasets. DoOR now gives reliable odorant-responses for nearly all *Drosophila* olfactory responding units, listing 693 odorants, for a total of 7381 data points.

Supplemental Material

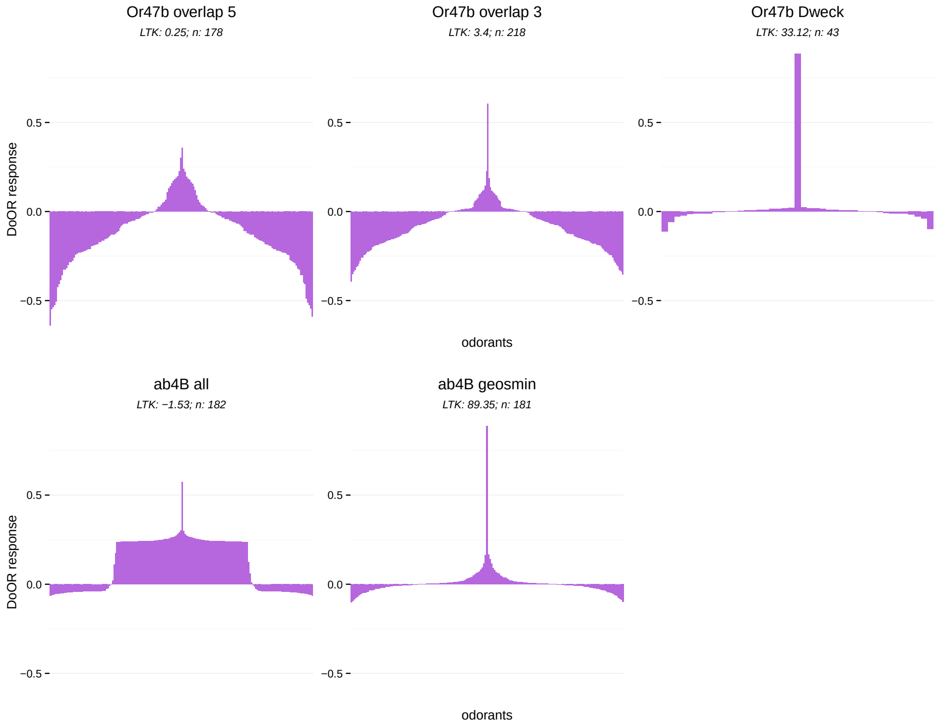

(a)

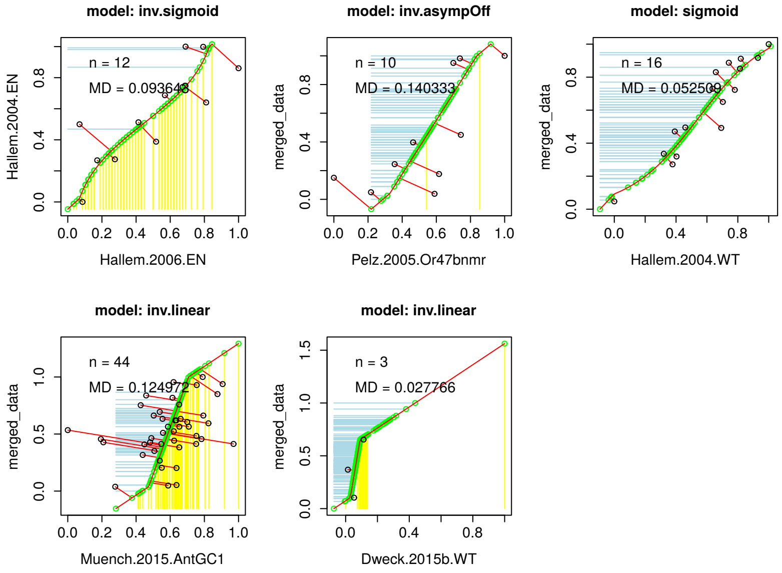

(b)

Figure S1. Related to Figure 3. Merging two narrowly tuned responding units with different merge-specifications. **a** *top row*, the response profiles of Or47b computed with different minimal overlap required between odorants and the Or47b response profile from Dweck *et al.*[1] With a minimal overlap of 5 the best ligand does not enter into DoOR, because the Dweck study has fewer common odorants with the other datasets. *bottom row*, the ab4B response profile in DoOR and the ab4B response profile when only merging studies that measure the best ligand geosmin (Stensmyr *et al.*[2] and the study at hand). Studies that do not contain the best ligand lead to a devaluation of the best ligand in the consensus dataset. **b** The step-wise merging process of the Or47b datasets with a minimal overlap of three odorants. Note how the odors that are outside the common range are plotted onto a straight line with slope 1. This is very clear in the last merging step, when `Dweck.2015.WT` is added, and the best ligand methyl laureate in this set is also projected onto the slope 1 line. *MD*, mean orthogonal distances between points and the best fitting function. Number of odorants in the dataset is given as *n*.

Table S1. Studies in DoOR. The table gives an overview over the studies that contributed to DoOR 2.0. Sums are given in the *bottom row*, values in parentheses are the corresponding values of DoOR 1.0

study	datasets	responding units	odorants
de Bruyne *et al.* 1999[3]	1	6	18
de Bruyne *et al.* 2001[4]	2	16	52
Dobritsa *et al.* 2003[5]	2	9	19
Stensmyr *et al.* 2003[6]	1	5	25
Hallem *et al.* 2004[7]	2	27	56
Goldman *et al.* 2005[8]	2	7	12
Kreher *et al.* 2005[9]	1	11	32
Pelz 2005[10]	3	2	79
Yao *et al.* 2005[11]	1	7	48
Hallem *et al.* 2006[12]	1	24	111
Pelz *et al.* 2006[13]	2	1	41
Kwon *et al.* 2007[14]	2	1	14
Nissler 2007[15]	2	1	111
Schmuker *et al.* 2007[16]	1	7	49
van der Goes van Naters *et al.* 2007[17]	1	2	7
Kreher *et al.* 2008[18]	1	23	30
Turner *et al.* 2009[19]	1	1	48
Galizia *et al.* 2010[20]	1	2	107
de Bruyne *et al.* 2010[21]	1	8	13
Marshall *et al.* 2010[22]	1	31	44
Montague *et al.* 2011[23]	1	21	28
Silbering *et al.* 2011[24]	3	15	172
Stensmyr *et al.* 2012[2]	1	2	103
Dweck *et al.* 2013[25]	1	1	474
Gabler *et al.* 2013[26]	1	10	22
Ronderos *et al.* 2014[27]	1	1	125
Dweck *et al.* 2015[28]	2	3	102
Dweck *et al.* 2015a[1]	1	4	43
Muench *et al.* 2015	2	5	114
**29 (18)**	**42 (27)**	**78 (62)**	**693 (226)**

Table S2. Related to Figure 1a: The sequence of responding units shown on the y axis. *score* indicates the number of odorants tested with a given responding unit.

responding unit	score	responding unit	score
Or19a	497	ac3A	95
Or7a	246	ac2A	84
Or10a	235	ab5B	82
Or22a	225	Gr21a.Gr63a	80
ab4B	221	Or94b	66
Or47b	218	Or94a	65
Or82a	204	ac1A	63
Or42b	201	Or45a	56
Or13a	191	Or22c	55
ac4	190	Or24a	55
Or59b	181	Or30a	55
Or92a	174	Or45b	55
ac1	172	Or49a	55
ac2	172	Or59a	55
ac3_noOr35a	172	Or74a	55
Or85b	169	Or85c	55
Or49b	164	Or59c	53
Or65a	163	Or46a	51
Or47a	161	Or85d	51
Or67c	161	pb2A	51
Or98a	161	ac2B	48
Or88a	157	Or67d	48
Or2a	150	ac1B	47
Or35a	149	ac2BC	44
Or71a	149	Or1a	30
Or33b	148	Or33a	30
Or67b	146	Ir31a	24
Or9a	144	Ir41a	24
Or43b	144	Ir75a	24
Or67a	127	Ir75d	24
Or83c	125	Ir76a	24
Or23a	115	Ir84a	24
Or43a	115	Ir92a	24
Or85a	114	Ir64a.DC4	24
Or85f	114	Ir64a.DP1m	24
Or69a	107	ac1BC	24
ab2B	101	Or33c	12
ac3B	98	Or85e	12
Or42a	96	Or22b	11

Table S3. Related to Figure 1a: The sequence of odorants shown on the x axis. *score* indicates the number of responding units a given odorant was tested in.

InChIKey	name	score
SFR	SFR	77
solvent	oil	76
XEKOWRVHYACXOJ-UHFFFAOYSA-N	ethyl acetate	74
HUMNYLRZRPPJDN-UHFFFAOYSA-N	benzaldehyde	67
PGMYKACGEOXYJE-UHFFFAOYSA-N	pentyl acetate	67
CATSNJVOTSVZJV-UHFFFAOYSA-N	2-heptanone	66
MBDOYVRWFFCFHM-SNAWJCMRSA-N	E2-hexenal	66
VSMOENVRRABVKN-UHFFFAOYSA-N	1-octen-3-ol	65
XBDQKXXYIPTUBI-UHFFFAOYSA-N	propanoic acid	65
HIGQPQRQIQDZMP-DHZHZOJOSA-N	geranyl acetate	64
QSJXEFYPDANLFS-UHFFFAOYSA-N	2,3-butanedione	64
OBNCKNCVKJNDBV-UHFFFAOYSA-N	ethyl butyrate	63
JHIVVAPYMSGYDF-UHFFFAOYSA-N	cyclohexanone	62
QWVGKYWNOKOFNN-UHFFFAOYSA-N	2-methylphenol	61
XLYOFNOQVPJJNP-UHFFFAOYSA-N	water	60
ZSIAUFGUXNUGDI-UHFFFAOYSA-N	1-hexanol	59
LRHPLDYGYMQRHN-UHFFFAOYSA-N	1-butanol	58
MLFHJEHSLIIPHL-UHFFFAOYSA-N	isopentyl acetate	58
KIDHWZJUCRJVML-UHFFFAOYSA-N	putrescine	57
OSWPMRLSEDHDFF-UHFFFAOYSA-N	methyl salicylate	55
VHUUQVKOLVNVRT-UHFFFAOYSA-N	ammonium hydroxide	55
FERIUCNNQQJTOY-UHFFFAOYSA-N	butyric acid	54
CURLTUGMZLYLDI-UHFFFAOYSA-N	carbon dioxide	52
ZWEHNKRNPOVVGH-UHFFFAOYSA-N	2-butanone	52
KWOLFJPFCHCOCG-UHFFFAOYSA-N	acetophenone	51
NMRPBPVERJPACX-UHFFFAOYSA-N	3-octanol	51
CSCPPACGZOOCGX-UHFFFAOYSA-N	acetone	50
DTUQWGWMVIHBKE-UHFFFAOYSA-N	phenylacetaldehyde	50
IWDCLRJOBJJRNH-UHFFFAOYSA-N	4-methylphenol	50
NBBJYMSMWIIQGU-UHFFFAOYSA-N	propanal	50
ZTQSAGDEMFDKMZ-UHFFFAOYSA-N	butanal	50
YKYONYBAUNKHLG-UHFFFAOYSA-N	propyl acetate	49
WRMNZCZEMHIOCP-UHFFFAOYSA-N	phenethyl alcohol	48
OWBTYPJTUOEWEK-UHFFFAOYSA-N	2,3-butanediol	47
QTBSBXVTEAMEQO-UHFFFAOYSA-N	acetic acid	47
UHEPJGULSIKKTP-UHFFFAOYSA-N	6-methyl-5-hepten-2-one	46
PHTQWCKDNZKARW-UHFFFAOYSA-N	3-methyl-butanol	44
ZYEMGPIYFIJGTP-UHFFFAOYSA-N	4-allyl-1,2-dimethoxybenzene	44
AOGQPLXWSUTHQB-UHFFFAOYSA-N	hexyl acetate	42
LHXDLQBQYFFVNW-OIBJUYFYSA-N	(1R)-(-)-fenchone	42
DKPFZGUDAPQIHT-UHFFFAOYSA-N	butyl acetate	41
FUZZWVXGSFPDMH-UHFFFAOYSA-N	hexanoic acid	41
HGBOYTHUEUWSSQ-UHFFFAOYSA-N	pentanal	41
JARKCYVAAOWBJS-UHFFFAOYSA-N	hexanal	41
WVDDGKGOMKODPV-UHFFFAOYSA-N	benzyl alcohol	41
UFLHIIWVXFIJGU-ARJAWSKDSA-N	Z3-hexenol	40
VHRGRCVQAFMJIZ-UHFFFAOYSA-N	cadaverine	40
RRAFCDWBNXTKKO-UHFFFAOYSA-N	eugenol	39
GWYFCOCPABKNJV-UHFFFAOYSA-N	isopentanoic acid	38
CDOSHBSSFJOMGT-UHFFFAOYSA-N	linalool	37
CZUGFKJYCPYHHV-UHFFFAOYSA-N	3-methylthio-1-propanol	36
GLZPCOQZEFWAFX-JXMROGBWSA-N	geraniol	36
HYBBIBNJHNGZAN-UHFFFAOYSA-N	furfural	36
KBPLFHHGFOOTCA-UHFFFAOYSA-N	1-octanol	36
RDOXTESZEPMUJZ-UHFFFAOYSA-N	4-methoxybenzene	36
XAPCMTMQBXLDBB-UHFFFAOYSA-N	hexyl butyrate	36
ZWRUINPWMLAQRD-UHFFFAOYSA-N	1-nonanol	36
AMQJEAYHLZJPGS-UHFFFAOYSA-N	1-pentanol	35
FBUKVWPVBMHYJY-UHFFFAOYSA-N	nonanoic acid	35
FKRCODPIKNYEAC-UHFFFAOYSA-N	ethyl propionate	35
MTZQAGJQAFMTAQ-UHFFFAOYSA-N	ethyl benzoate	35
NQPDZGIKBAWPEJ-UHFFFAOYSA-N	pentanoic acid	35
XNLICIUVMPYHGG-UHFFFAOYSA-N	2-pentanone	35
HRHOWZHRCRZVCU-WAYWQWQTSA-N	E2-hexenyl acetate	34
ICMAFTSLXCXHRK-UHFFFAOYSA-N	ethyl pentanoate	34
JBFHTYHTHYHCDJ-UHFFFAOYSA-N	gamma-hexalactone	34
KQNPFQTWMSNSAP-UHFFFAOYSA-N	isobutyric acid	34
MNWFXJYAOYHMED-UHFFFAOYSA-N	heptanoic acid	34
RWRDLPDLKQPQOW-UHFFFAOYSA-N	pyrrolidine	34
WWZKQHOCKIZLMA-UHFFFAOYSA-N	octanoic acid	34
ZCHHRLHTBGRGOT-SNAWJCMRSA-N	E2-hexenol	34
IKHGUXGNUITLKF-UHFFFAOYSA-N	acetaldehyde	33
UXFSPRAGHGMRSQ-UHFFFAOYSA-N	2-isobutyl-3-methoxypyrazine	33
CHWNEIVBYREQRF-UHFFFAOYSA-N	4-ethylguaiacol	32
GYHFUZHODSMOHU-UHFFFAOYSA-N	nonanal	32
KXKVLQRXCPHEJC-UHFFFAOYSA-N	methyl acetate	32
QCCDLTOVEPVEJK-UHFFFAOYSA-N	phenylacetone	32
QMVPMAAFGQKVCJ-SNVBAGLBSA-N	beta-citronellol	32
YIWUKEYIRIRTPP-UHFFFAOYSA-N	2-Ethyl-1-hexanol	32
BDZBKCUKTQZUTL-UHFFFAOYSA-N	Triethyl phosphite	31
CRVGTESFCCXCTH-UHFFFAOYSA-N	Methyldiethanolamine	31
GRYLNZFGIOXLOG-UHFFFAOYSA-N	Nitric Acid	31
MHAJPDPJQMAIIY-UHFFFAOYSA-N	Hydrogen peroxide	31
OMSUIQOIVADKIM-UHFFFAOYSA-N	ethyl 3-hydroxybutyrate	31
RWSOTUBLDIXVET-UHFFFAOYSA-N	Sulfur	31
WUOACPNHFRMFPN-SECBINFHSA-N	alpha-terpineol	31
YLYBTZIQSIBWLI-UHFFFAOYSA-N	octyl acetate	31
ZHNUHDYFZUAESO-UHFFFAOYSA-N	Formamide	31
BBMCTIGTTCKYKF-UHFFFAOYSA-N	1-heptanol	30
JYVLIDXNZAXMDK-UHFFFAOYSA-N	2-pentanol	30
LFQSCWFLJHTTHZ-UHFFFAOYSA-N	ethanol	30
NPNUFJAVOOONJE-GFUGXAQUSA-N	(-)-trans-caryophyllene	30
NUKZAGXMHTUAFE-UHFFFAOYSA-N	methyl hexanoate	30
OKKJLVBELUTLKV-UHFFFAOYSA-N	methanol	30
QPJVMBTYPHYUOC-UHFFFAOYSA-N	methyl benzoate	30
SHZIWNPUGXLXDT-UHFFFAOYSA-N	ethyl hexanoate	30
BDERNNFJNOPAEC-UHFFFAOYSA-N	1-propanol	29
DKMROQRQHGEIOW-UHFFFAOYSA-N	diethyl succinate	29
DWCLXOREGBLXTD-UHFFFAOYSA-N	2,3-Dimethyl-2,3-dinitrobutane	29
GJRQTCIYDGXPES-UHFFFAOYSA-N	isobutyl acetate	29
GRWFGVWFFZKLTI-UHFFFAOYSA-N	alpha-pinene	29
HMWPNDNFTFSCEB-UHFFFAOYSA-N	Hexamethylene triperoxide-diamine	29
JGHZJRVDZXSNKQ-UHFFFAOYSA-N	methyl octanoate	29
LYGJENNIWJXYER-UHFFFAOYSA-N	Nitromethane	29
NCDCLPBOMHPFCV-UHFFFAOYSA-N	hexyl hexanoate	29
UAHWPYUMFXYFJY-UHFFFAOYSA-N	beta-myrcene	29
UUIQMZJEGPQKFD-UHFFFAOYSA-N	methyl butyrate	29
VKYKSIONXSXAKP-UHFFFAOYSA-N	Hexamine	29
WFDIJRYMOXRFFG-UHFFFAOYSA-N	Acetic anhydride	29
WGSVFWFSJDAYBM-BQYQJAHWSA-N	1-phenyl-2-nitropropene	29
YYZUSRORWSJGET-UHFFFAOYSA-N	ethyl octanoate	29
ZMQAAUBTXCXRIC-UHFFFAOYSA-N	Safrole	29
ZTLXICJMNFREPA-UHFFFAOYSA-N	Triacetone triperoxide	29
AKNUHUCEWALCOI-UHFFFAOYSA-O	Ethyldiethanolamine	28
BAZAXWOYCMUHIX-UHFFFAOYSA-M	Sodium perchlorate	28
BQOFWKZOCNGFEC-UHFFFAOYSA-N	3-carene	28
BRHDDEIRQPDPMG-UHFFFAOYSA-N	linalool oxide	28
FHIVAFMUCKRCQO-UHFFFAOYSA-N	Diazinon	28
JVTAAEKCZFNVCJ-UHFFFAOYSA-N	lactic acid	28
KBEBGUQPQBELIU-CMDGGOBGSA-N	ethyl cinnamate	28
LCTONWCANYUPML-UHFFFAOYSA-N	pyruvic acid	28
MOYAFQVGZZPNRA-UHFFFAOYSA-N	terpinolene	28
OBETXYAYXDNJHR-UHFFFAOYSA-N	2-ethylhexanoic acid	28
OSPSWZSRKYCQPF-UHFFFAOYSA-N	Diethyl phosphite	28
PEDCQBHIVMGVHV-UHFFFAOYSA-N	glycerol	28
QMMFVYPAHWMCMS-UHFFFAOYSA-N	dimethyl sulfide	28
RDYMFSUJUZBWLH-UHFFFAOYSA-N	Endosulfan	28
RGXWDWUGBIJHDO-UHFFFAOYSA-N	ethyl decanoate	28
SNIOPGDIGTZGOP-UHFFFAOYSA-N	Nitroglycerin	28
SPSSULHKWOKEEL-UHFFFAOYSA-N	2,4,6-Trinitrotoluene	28
TZRXHJWUDPFEEY-UHFFFAOYSA-N	Pentaerythritol tetranitrate	28
XTFIVUDBNACUBN-UHFFFAOYSA-N	Cyclotrimethylenetrinitramine	28
YEJRWHAVMIAJKC-UHFFFAOYSA-N	gamma-butyrolactone	28
YLMGFJXSLBMXHK-UHFFFAOYSA-M	Potassium perchlorate	28
ZFDIRQKJPRINOQ-HWKANZROSA-N	ethyl trans-2-butenoate	28
ZKQDCIXGCQPQNV-UHFFFAOYSA-N	Chlorine	28
XMGQYMWWDOXHJM-UHFFFAOYSA-N	limonene	27
ZCHHRLHTBGRGOT-PLNGDYQASA-N	Z2-hexenol	27
GAEKPEKOJKCEMS-UHFFFAOYSA-N	gamma-valerolactone	26
GLZPCOQZEFWAFX-YFHOEESVSA-N	nerol	26
HFPZCAJZSCWRBC-UHFFFAOYSA-N	p-cymene	26
KYQCOXFCLRTKLS-UHFFFAOYSA-N	pyrazine	26
MQWCXKGKQLNYQG-UHFFFAOYSA-N	4-methylcyclohexanol	26
BDAGIHXWWSANSR-UHFFFAOYSA-N	methanoic acid	25
FAMPSKZZVDUYOS-HRGUGZIWSA-N	alpha-humulene	25
LZCLXQDLBQLTDK-UHFFFAOYSA-N	ethyl lactate	25
MDHYEMXUFSJLGV-UHFFFAOYSA-N	phenethyl acetate	25
SIKJAQJRHWYJAI-UHFFFAOYSA-N	indole	25
UFLHIIWVXFIJGU-ONEGZZNKSA-N	E3-hexenol	25
VHVMXWZXFBOANQ-UHFFFAOYSA-N	1-penten-3-ol	25
ASUAYTHWZCLXAN-UHFFFAOYSA-N	3-methyl-2-buten-1-ol	24
GHBSPIPJMLAMEP-UHFFFAOYSA-N	delta-decalactone	24
HGINCPLSRVDWNT-UHFFFAOYSA-N	2-propenal	24
IFYYFLINQYPWGJ-UHFFFAOYSA-N	gamma-decalactone	24
IPBFYZQJXZJBFQ-UHFFFAOYSA-N	gamma-octalactone	24
OYHQOLUKZRVURQ-HZJYTTRNSA-N	linoleic acid	24
QGZKDVFQNNGYKY-UHFFFAOYSA-N	ammonia	24
WBJINCZRORDGAQ-UHFFFAOYSA-N	ethyl methanoate	24
WTARULDDTDQWMU-UHFFFAOYSA-N	beta-pinene	24
XMGQYMWWDOXHJM-JTQLQIEISA-N	(R)-(+)-limonene	24
KSMVZQYAVGTKIV-UHFFFAOYSA-N	decanal	23
NUJGJRNETVAIRJ-UHFFFAOYSA-N	octanal	23
CAWHJQAVHZEVTJ-UHFFFAOYSA-N	2-Methylpyrazine	22
FINHMKGKINIASC-UHFFFAOYSA-N	2,3,5,6-Tetramethylpyrazine	22
GZXXANJCCWGCSV-UHFFFAOYSA-N	2,3-Diethylpyrazine	22
KVFIJIWMDBAGDP-UHFFFAOYSA-N	2-Ethylpyrazine	22
LCZUOKDVTBMCMX-UHFFFAOYSA-N	2,5-Dimethylpyrazine	22
OXQOBQJCDNLAPO-UHFFFAOYSA-N	2,3-Dimethylpyrazine	22
QQZOPKMRPOGIEB-UHFFFAOYSA-N	2-hexanone	22
VKJIAEQRKBQLLA-UHFFFAOYSA-N	2-Methoxy-3-methylpyrazine	22
WKSXRWSOSLGSTN-UHFFFAOYSA-N	2-Methoxypyrazine	22
BOFLOMVCGPWPQC-UHFFFAOYSA-N	2-Ethyl-3,(5 or 6)-dimethylpyrazine	21
DBZAKQWXICEWNW-UHFFFAOYSA-N	2-Acetylpyrazine	21
DPCILIMHENXHQX-UHFFFAOYSA-N	2-Ethyl-3-methoxypyrazine	21
FXHGMKSSBGDXIY-UHFFFAOYSA-N	heptanal	21
GELVZYOEQVJIRR-UHFFFAOYSA-N	2-Chloropyrazine	21
HCRBXQFHJMCTLF-UHFFFAOYSA-N	ethyl 2-methylbutanoate	21
HJFZAYHYIWGLNL-UHFFFAOYSA-N	2,6-Dimethylpyrazine	21
IAEGWXHKWJGQAZ-UHFFFAOYSA-N	2,3,5-Trimethylpyrazine	21
LNIMMWYNSBZESE-UHFFFAOYSA-N	2-Ethyl-3-methylpyrazine	21
MHVIQJDXHDKUAM-UHFFFAOYSA-N	2-Ethyl-5(6)-methylpyrazine	21
PPJSYGVFDJEMRP-UHFFFAOYSA-N	2-Acetyl-3-ethylpyrazine	21
QKVWBAMZPUHCMO-UHFFFAOYSA-N	Pyrazineethanethiol	21
QMQDJVIJVPEQHE-UHFFFAOYSA-N	2-methoxy-3-(1-methylpropyl)pyrazine	21
QUNOTZOHYZZWKQ-UHFFFAOYSA-N	2-Acetyl-3-methylpyrazine	21
UCGOSAWBWFUKDT-UHFFFAOYSA-N	2-Acetyl-3,5(or 6)-dimethylpyrazine	21
VKCYHJWLYTUGCC-UHFFFAOYSA-N	nonanone	21
YZEFQPIMXZVPKP-UHFFFAOYSA-N	5H-5-Methyl-6, 7-dihydrocyclopenta[b]pyrazine	21
ZHMIODDNZRIENW-UHFFFAOYSA-N	2-isobutyl-3-Methylpyrazine	21
HZAXFHJVJLSVMW-UHFFFAOYSA-N	ethanolamine	20
MWKFXSUHUHTGQN-UHFFFAOYSA-N	decanol	20
WEEGYLXZBRQIMU-WAAGHKOSSA-N	1,8-cineole	20
WTWBUQJHJGUZCY-UHFFFAOYSA-N	4-isopropylbenzaldehyde	20
ZPVFWPFBNIEHGJ-UHFFFAOYSA-N	2-octanone	20
ROSDSFDQCJNGOL-UHFFFAOYSA-N	dimethylamine	19
BHHGXPLMPWCGHP-UHFFFAOYSA-N	2-phenylethylamine	18
QUSNBJAOOMFDIB-UHFFFAOYSA-N	Ethyl amine	18
WLJVXDMOQOGPHL-UHFFFAOYSA-N	Phenylacetic acid	18
ALVPFGSHPUPROW-UHFFFAOYSA-N	dipropyldisulphide	17
HPXRVTGHNJAIIH-UHFFFAOYSA-N	cyclohexanol	17
JUJWROOIHBZHMG-UHFFFAOYSA-N	Pyridine	17
KDVFRMMRZOCFLS-UHFFFAOYSA-N	2-oxovaleric acid	17
SUSQOBVLVYHIEX-UHFFFAOYSA-N	benzyl cyanide	17
LVBXEMGDVWVTGY-VOTSOKGWSA-N	E2-octenal	16
WTEVQBCEXWBHNA-JXMROGBWSA-N	citral	16
ATHGHQPFGPMSJY-UHFFFAOYSA-N	spermidine	15
SUVIGLJNEAMWEG-UHFFFAOYSA-N	propanethiol	15
ZXEKIIBDNHEJCQ-UHFFFAOYSA-N	isobutyl alcohol	15
HQABUPZFAYXKJW-UHFFFAOYSA-N	butylamine	14
QSZKEDWVAOAFQY-HJWRWDBZSA-N	11-cis Vaccenyl Acetate	14
ROWKJAVDOGWPAT-UHFFFAOYSA-N	3-hydroxy-2-butanone	14
ULDHMXUKGWMISQ-SECBINFHSA-N	(R)-(-)-carvone	14
ULDHMXUKGWMISQ-VIFPVBQESA-N	(S)-(+)-carvone	14
BJIOGJUNALELMI-ONEGZZNKSA-N	iso-eugenol	13
IMNFDUFMRHMDMM-UHFFFAOYSA-N	heptane	13
NPFVOOAXDOBMCE-PLNGDYQASA-N	Z3-hexenyl acetate	13
QNVRIHYSUZMSGM-UHFFFAOYSA-N	2-hexanol	13
QUKGYYKBILRGFE-UHFFFAOYSA-N	benzyl acetate	13
XUPYJHCZDLZNFP-UHFFFAOYSA-N	butyl butanoate	13
GRWFGVWFFZKLTI-RKDXNWHRSA-N	(+)-alpha-pinene	12
LABTWGUMFABVFG-ARJAWSKDSA-N	3-penten-2-one	12
LYRIITRHDCNUHV-UHFFFAOYSA-N	ethyl 3-hydroxyhexanoate	12
CETWDUZRCINIHU-ZETCQYMHSA-N	(2S)-heptan-2-ol	11
CRDAMVZIKSXKFV-YFVJMOTDSA-N	E,E-farnesol	11
VUPBIVVRPJDWNW-FNORWQNLSA-N	isopropyl tiglate	11
WJYIASZWHGOTOU-UHFFFAOYSA-N	heptylamine	11
YSNWHRKJEKWJNY-UHFFFAOYSA-N	ethyl 3-methylsulfanylpropanoate	11
BHIWKHZACMWKOJ-UHFFFAOYSA-N	Methyl 2-methylpropanoate	10
BMFVGAAISNGQNM-UHFFFAOYSA-N	isoamylamine	10
BTMVHUNTONAYDX-UHFFFAOYSA-N	butyl propanoate	10
FWZUNOYOVVKUNF-UHFFFAOYSA-N	prop-2-enyl acetate	10
GMEONFUTDYJSNV-UHFFFAOYSA-N	Ethyl 4-oxopentanoate	10
JIGUQPWFLRLWPJ-UHFFFAOYSA-N	ethyl prop-2-enoate	10
MEIRRNXMZYDVDW-MQQKCMAXSA-N	hexa-2,4-dien-1-ol	10
NEHNMFOYXAPHSD-JTQLQIEISA-N	(S)-(-)-citronellal	10
OAPHLAAOJMTMLY-GQCTYLIASA-N	ethyl 2-methyl-2E-butenoate	10
PFCHFHIRKBAQGU-UHFFFAOYSA-N	3-hexanone	10
QNVRIHYSUZMSGM-ZCFIWIBFSA-N	(2R)-hexan-2-ol	10
RJUFJBKOKNCXHH-UHFFFAOYSA-N	methyl propionate	10
VLKZOEOYAKHREP-UHFFFAOYSA-N	hexane	10
WYACBZDAHNBPPB-UHFFFAOYSA-N	Diethyl oxalate	10
ACCRBMDJCPPJDX-UHFFFAOYSA-N	methyl 3-hydroxy hexanoate	9
BKIMMITUMNQMOS-UHFFFAOYSA-N	nonane	9
CETWDUZRCINIHU-UHFFFAOYSA-N	2-heptanol	9
GSCLMSFRWBPUSK-UHFFFAOYSA-N	beta-butyrolactone	9
HNZUNIKWNYHEJJ-FMIVXFBMSA-N	geranyl acetone	9
KMRMUZKLFIEVAO-UHFFFAOYSA-N	(1R)-(-)-myrtenal	9
LCHYEKKJCUJAKN-UHFFFAOYSA-N	2-propylphenol	9
NZGWDASTMWDZIW-MRVPVSSYSA-N	(R)-(+)-pulegon	9
OMSUIQOIVADKIM-RXMQYKEDSA-N	ethyl (R)-(-)-3-hydroxybutanoate	9
OMSUIQOIVADKIM-YFKPBYRVSA-N	ethyl (S)-(+)-3-hydroxybutanoate	9
PPXUHEORWJQRHJ-UHFFFAOYSA-N	ethyl isovalerate	9
PSQYTAPXSHCGMF-BQYQJAHWSA-N	beta-ionone	9
RGZSQWQPBWRIAQ-CABCVRRESA-N	alpha-bisabolol	9
RHLVCLIPMVJYKS-UHFFFAOYSA-N	3-octanone	9
RUVINXPYWBROJD-ONEGZZNKSA-N	4-propenyl anisole	9
TVMXDCGIABBOFY-UHFFFAOYSA-N	octane	9
USMNOWBWPHYOEA-MRTMQBJTSA-N	(-)-alpha-thujone	9
UZFLPKAIBPNNCA-BQYQJAHWSA-N	alpha-ionone	9
XHIUFYZDQBSEMF-UHFFFAOYSA-N	2-methyl butyl acetate	9
ZRSNZINYAWTAHE-UHFFFAOYSA-N	4-methoxybenzaldehyde	9
BVOSSZSHBZQJOI-UHFFFAOYSA-N	1-hexen-3-ol	8
FFOPEPMHKILNIT-UHFFFAOYSA-N	isopropyl butyrate	8
GEWDNTWNSAZUDX-SNAWJCMRSA-N	methyl jasmonate	8
HXDOZKJGKXYMEW-UHFFFAOYSA-N	4-ethylphenol	8
IEMMBWWQXVXBEU-UHFFFAOYSA-N	acetyl furan	8
IXQGCWUGDFDQMF-UHFFFAOYSA-N	2-ethylphenol	8
JFDZBHWFFUWGJE-UHFFFAOYSA-N	benzonitrile	8
KLSLBUSXWBJMEC-UHFFFAOYSA-N	4-propylphenol	8
OUGPMNMLWKSBRI-UHFFFAOYSA-N	hexyl methanoate	8
VLSVVMPLPMNWBH-UHFFFAOYSA-N	gamma-propyl-gamma-butyrolactone	8
WOFPPJOZXUTRAU-UHFFFAOYSA-N	4-octanol	8
YYJWBYNQJLBIGS-PLNGDYQASA-N	methyl tiglate	8
ZARFDQHJMNVNLE-WEVVVXLNSA-N	isoamyl tiglate	8
ZCZSIDMEHXZRLG-UHFFFAOYSA-N	heptyl acetate	8
GQKZRWSUJHVIPE-UHFFFAOYSA-N	sec-amyl acetate	7
LHGVFZTZFXWLCP-UHFFFAOYSA-N	2-hydroxy-anisole	7
NDFKTBCGKNOHPJ-AATRIKPKSA-N	E2-heptenal	7
RFFOTVCVTJUTAD-UHFFFAOYSA-N	1,4-cineol	7
UWKAYLJWKGQEPM-UHFFFAOYSA-N	linalyl acetate	6
YHQGMYUVUMAZJR-UHFFFAOYSA-N	alpha-terpinene	6
YOMSJEATGXXYPX-UHFFFAOYSA-N	2-methoxy-4-vinyl phenol	6
YXFVVABEGXRONW-UHFFFAOYSA-N	toluene	6
AMTITFMUKRZZEE-WAYWQWQTSA-N	cis-11-hexadecenal	5
BGHCVCJVXZWKCC-UHFFFAOYSA-N	tetradecane	5
BJQWYEJQWHSSCJ-UHFFFAOYSA-N	heptacosane	5
CDOSHBSSFJOMGT-JTQLQIEISA-N	(-)-linalool	5
DCAYPVUWAIABOU-UHFFFAOYSA-N	hexadecane	5
DPBLXKKOBLCELK-UHFFFAOYSA-N	pentylamine	5
FIGVVZUWCLSUEI-UHFFFAOYSA-N	tricosane	5
FNAZRRHPUDJQCJ-UHFFFAOYSA-N	heneicosan	5
GETQZCLCWQTVFV-UHFFFAOYSA-N	trimethylamine	5
HIGQPQRQIQDZMP-FLIBITNWSA-N	cis-3,7-dimethyl-2,6-octadien-1-yl acetate	5
HMSWAIKSFDFLKN-UHFFFAOYSA-N	hexacosane	5
HOWGUJZVBDQJKV-UHFFFAOYSA-N	docosane	5
HUAZGNHGCJGYNP-UHFFFAOYSA-N	propyl butanoate	5
IPCSVZSSVZVIGE-UHFFFAOYSA-N	palmitic acid	5
KIPCKEJKGCXRGA-UHFFFAOYSA-N	2-ethylfenchol	5
MWOOGOJBHIARFG-UHFFFAOYSA-N	4hydroxy-3-methoxybenzaldehyde	5
NOOLISFMXDJSKH-KXUCPTDWSA-N	menthol	5
POOSGDOYLQNASK-UHFFFAOYSA-N	tetracosane	5
POULHZVOKOAJMA-UHFFFAOYSA-N	dodecanoic acid	5
RLSSMJSEOOYNOY-UHFFFAOYSA-N	3-methylphenol	5
TUNFSRHWOTWDNC-UHFFFAOYSA-N	myristic acid	5
WHUUTDBJXJRKMK-UHFFFAOYSA-N	glutamate	5
WTARULDDTDQWMU-IUCAKERBSA-N	(-)-beta-pinene	5
XMGQYMWWDOXHJM-SNVBAGLBSA-N	L-limonen	5
YKNWIILGEFFOPE-UHFFFAOYSA-N	pentacosane	5
ZAZKJZBWRNNLDS-UHFFFAOYSA-N	methyl myristate	5
ZOCHHNOQQHDWHG-UHFFFAOYSA-N	3-hexanol	5
ZYURHZPYMFLWSH-UHFFFAOYSA-N	octacosane	5
AISQJRLSHDWVGU-UHFFFAOYSA-N	2-methyl docosane	4
BEBPORIYFVRVCP-UHFFFAOYSA-N	2-methyl hexacosane	4
CBFCDTFDPHXCNY-UHFFFAOYSA-N	Eicosane	4
DHMQDGOQFOQNFH-UHFFFAOYSA-N	glycine	4
DUFKCOQISQKSAV-UHFFFAOYSA-N	propylene glycol	4
FEXQDZTYJVXMOS-UHFFFAOYSA-N	Isopropyl benzoate	4
FLIACVVOZYBSBS-UHFFFAOYSA-N	methyl palmitate	4
FQMZVFJYMPNUCT-YRNVUSSQSA-N	geranyl formate	4
GQEZCXVZFLOKMC-UHFFFAOYSA-N	hexadecene	4
HMNKTRSOROOSPP-UHFFFAOYSA-N	3-ethylphenol	4
HTUIWRWYYVBCFT-UHFFFAOYSA-N	propyl hexanoate	4
HVGZQCSMLUDISR-UHFFFAOYSA-N	Phenylethyl propionate	4
IGGUPRCHHJZPBS-UHFFFAOYSA-N	Nonacosane	4
IGOWHGRNPLFNDJ-ZPHPHTNESA-N	(Z)-9-tricosene	4
IIYFAKIEWZDVMP-UHFFFAOYSA-N	Tridecane	4
IRYCVIRCWSSJOW-SQFISAMPSA-N	(Z)-7-tricosene	4
ISWSIDIOOBJBQZ-UHFFFAOYSA-N	phenol	4
KJSVCIRMHUQNGQ-ADYYPQGGSA-N	(Z,Z)-7,11-octacosadiene	4
KWUWRILZYFCPRI-ADYYPQGGSA-N	(Z,Z)-7,11-nonacosadiene	4
LQERIDTXQFOHKA-UHFFFAOYSA-N	Nonadecane	4
MLLAPOCBLWUFAP-UHFFFAOYSA-N	Isoamyl benzoate	4
MWORWWXTNWRHAD-UHFFFAOYSA-N	5-methyl tricosane	4
NDJKXXJCMXVBJW-UHFFFAOYSA-N	Heptadecane	4
NGDNVOAEIVQRFH-UHFFFAOYSA-N	2-nonanol	4
PAFZNILMFXTMIY-UHFFFAOYSA-N	cyclohexylamine	4
PFNFFQXMRSDOHW-UHFFFAOYSA-N	spermine	4
PJVWXEURPGIPNW-ADYYPQGGSA-N	(Z,Z)-7,11-pentacosadiene	4
PQLMXFQTAMDXIZ-UHFFFAOYSA-N	Isoamyl butyrate	4
QDUXDCXILAPLAG-UHFFFAOYSA-N	N-methylpiperidine	4
QMVPMAAFGQKVCJ-JTQLQIEISA-N	(-)-beta-citronellol	4
RJYQFYALHBHYMG-ADYYPQGGSA-N	(Z,Z)-7,11-heptacosadiene	4
RORWYUWDGGVNRJ-SQFISAMPSA-N	(Z)-7-pentacosene	4
RPRPDTXKGSIXMD-UHFFFAOYSA-N	butyl hexanoate	4
RZJRJXONCZWCBN-UHFFFAOYSA-N	Octadecane	4
SATCULPHIDQDRE-UHFFFAOYSA-N	piperonal	4
SECPZKHBENQXJG-FPLPWBNLSA-N	Palmitoleic acid	4
SYDNSSSQVSOXTN-UHFFFAOYSA-N	4-methyl-2-nitrophenol	4

Table S4. Table S3 *continued*

InChIKey	name	score
TYEYBOSBBBHJIV-UHFFFAOYSA-N	2-oxobutyric acid	4
UDEWPOVQBGFNGE-UHFFFAOYSA-N	propyl benzoate	4
ULRDOMATUJWTEF-UHFFFAOYSA-N	7-methyl tricosane	4
UQDUPQYQJKYHQI-UHFFFAOYSA-N	methyl laurate	4
VEXZGXHMUGYJMC-UHFFFAOYSA-N	hydrogen chloride	4
VTBOTOBFGSVRMA-UHFFFAOYSA-N	1-methylcyclohexanol	4
WEGYGNROSJDEIW-UHFFFAOYSA-N	3-acetylpyridine	4
XDEGQMQKHFPBEW-VMPITWQZSA-N	Isobutyl tiglate	4
XVSZRAWFCDHCBP-UHFFFAOYSA-N	Isopentyl hexanoate	4
YCOZIPAWZNQLMR-UHFFFAOYSA-N	Pentadecane	4
YGCGPCUUTAKOEU-UHFFFAOYSA-N	2-methyl octacosane	4
YNQLUTRBYVCPMQ-UHFFFAOYSA-N	ethylbenzene	4
YNQOGIZOCQEUJR-UHFFFAOYSA-N	2-methyl tetracosane	4
ZOIRKXLFEHOVER-UHFFFAOYSA-N	Isopropyl isopentanoate	4
HPNMFZURTQLUMO-UHFFFAOYSA-N	Diethylamine	3
JLPUXFOGCDVKGO-GRYCIOLGSA-N	geosmin	3
JMMWKPVZQRWMSS-UHFFFAOYSA-N	Isopropyl acetate	3
KGEKLUUHTZCSIP-HOSYDEDBSA-N	(-) bornyl acetate	3
LFYXNXGVLGKVCJ-UHFFFAOYSA-N	2-methylisoborneol	3
LGTLDEUQCOJGFP-WAYWQWQTSA-N	Cis-3-hexenyl propionate	3
MMOXZBCLCQITDF-UHFFFAOYSA-N	DEET	3
NFLGAXVYCFJBMK-BDAKNGLRSA-N	(-)-menthone	3
NPFVOOAXDOBMCE-SNAWJCMRSA-N	Trans-3-hexenyl acetate	3
OCAIYHCKLADPEG-UHFFFAOYSA-N	Isopropyl pentanoate	3
PCWGTDULNUVNBN-UHFFFAOYSA-N	4-methyl pentanol	3
QEBNYNLSCGVZOH-UHFFFAOYSA-N	valencene	3
RSDDTPVXLMVLQE-UHFFFAOYSA-N	hexyl isovalerate	3
SESFRYSPDFLNCH-UHFFFAOYSA-N	benzyl benzoate	3
XAOGXQMKWQFZEM-UHFFFAOYSA-N	Isoamyl propionate	3
XXIKYCPRDXIMQM-UHFFFAOYSA-N	Prenyl acetate	3
ZKPTYCJWRHHBOW-UHFFFAOYSA-N	Acetoin acetate	3
ALSTYHKOOCGGFT-KTKRTIGZSA-N	oleyl alcohol	2
BCOXBEHFBZOJJZ-ARJAWSKDSA-N	cis-3-hexenyl benzoate	2
BHJHPYFAYGAPLS-UHFFFAOYSA-N	guaiacol acetate	2
BLRHMMGNCXNXJL-UHFFFAOYSA-N	1-methylindole	2
CCEFMUBVSUDRLG-XNWIYYODSA-N	(+)-limonene oxide	2
CXENHBSYCFFKJS-VDQVFBMKSA-N	farnesene	2
DFYRUELUNQRZTB-UHFFFAOYSA-N	acetovanillone	2
DGVVWUTYPXICAM-UHFFFAOYSA-N	2-mercaptoethanol	2
DKIDEFUBRARXTE-UHFFFAOYSA-N	3-mercaptopropanoate	2
DNIAPMSPPWPWGF-UHFFFAOYSA-N	1,2-propanediol	2
FGKJLKRYENPLQH-UHFFFAOYSA-N	4-methylvaleric acid	2
FUGYGGDSWSUORM-UHFFFAOYSA-N	4-vinylphenol	2
GDWRKZLROIFUML-UHFFFAOYSA-N	4-phenyl-2-butanol	2
GYGAZRPDUOHMAF-KHPPLWFESA-N	oleyl acetate	2
HRGPYCVTDOECMG-WALBABNVSA-N	methyl cedryl ether	2
IHPKGUQCSIINRJ-CSKARUKUSA-N	ocimene	2
JDQVBGQWADMTAM-UHFFFAOYSA-N	phenethyl isobutyrate	2
KGEKLUUHTZCSIP-UHFFFAOYSA-N	bornyl acetate	2
KJPRLNWUNMBNBZ-QPJJXVBHSA-N	3-phenyl-2E-propenal	2
KYZHGEFMXZOSJN-UHFFFAOYSA-N	isobutyl benzoate	2
LZWFXVJBIZIHCH-UHFFFAOYSA-N	1-octen-3-yl butyrate	2
NEHNMFOYXAPHSD-SNVBAGLBSA-N	(R)-(+)-Citronellal	2
NGAZZOYFWWSOGK-UHFFFAOYSA-N	3-heptanone	2
OALYTRUKMRCXNH-QMMMGPOBSA-N	gamma-pentyl-gamma-butyrolactone	2
OGLDWXZKYODSOB-SNVBAGLBSA-N	alpha-phellandrene	2
PVVODBCDJBGMJL-CMDGGOBGSA-N	trans-methyl vaccenate	2
PXIKRTCSSLJURC-UHFFFAOYSA-N	2-methoxy-4-propyl phenol	2
QAIPRVGONGVQAS-RQOWECAXSA-N	caffeic acid	2
QKNZNUNCDJZTCH-UHFFFAOYSA-N	n-pentyl benzoate	2
SMQUZDBALVYZAC-UHFFFAOYSA-N	salicyl aldehyde	2
UWHZIFQPPBDJPM-FPLPWBNLSA-N	cis-vaccenic acid	2
VGMJYYDKPUPTID-UHFFFAOYSA-N	4-ethylresorcinol	2
VONGZNXBKCOUHB-UHFFFAOYSA-N	benzyl butyrate	2
WNJSKZBEWNVKGU-UHFFFAOYSA-N	phenylacetaldehyde dimethyl acetal	2
WRFZKAGPPQGDDQ-UHFFFAOYSA-N	amyl hexanoate	2
XCYJPXQACVEIOS-UHFFFAOYSA-N	m-cymene	2
XMLQWXUVTXCDDL-FPLPWBNLSA-N	cis-vaccenyl alcohol	2
XPFVYQJUAUNWIW-UHFFFAOYSA-N	furfuryl alcohol	2
XSIFPSYPOVKYCO-UHFFFAOYSA-N	butyl benzoate	2
YKFLAYDHMOASIY-UHFFFAOYSA-N	gamma-terpinene	2
YZJCDVRXBOPXSQ-UHFFFAOYSA-N	benzyl valerate	2
ZCTQGTTXIYCGGC-UHFFFAOYSA-N	benzyl salicylate	2
ZFRKQXVRDFCRJG-UHFFFAOYSA-N	skatole	2
AFFLGGQVNFXPEV-UHFFFAOYSA-N	1-decene	1
AIQLNKITFBJPFO-WAYWQWQTSA-N	cis-3-hexenyl 3-methylbutanoate	1
AJKVQEKCUACUMD-UHFFFAOYSA-N	2-acetylpyridine	1
ARXJGSRGQADJSQ-UHFFFAOYSA-N	1-methoxy-2-propanol	1
AUKVIBNBLXQNIZ-UHFFFAOYSA-N	2,4-dimethylheptane	1
AXISYYRBXTVTFY-UHFFFAOYSA-N	isopropyl myristate	1
AXIUBBVSOWPLDA-UHFFFAOYSA-N	2-ethyl-p-xylene	1
AYWJSCLAAPJZEF-UHFFFAOYSA-N	butyl isovalerate	1
AZQWKYJCGOJGHM-UHFFFAOYSA-N	p-benzoquinone	1
BAMPVSWRQZNDQC-UHFFFAOYSA-N	2,4,5-Trimethylthiazole	1
BATOPAZDIZEVQF-MQQKCMAXSA-N	trans,trans-2,4-hexadienal	1
BAUZLFKYYIVGPM-UHFFFAOYSA-N	cyclononanone	1
BAVONGHXFVOKBV-RGURZIINSA-N	(-)-Carveol	1
BGTOWKSIORTVQH-UHFFFAOYSA-N	cyclopentanone	1
BHNHHSOHWZKFOX-UHFFFAOYSA-N	2-methylindole	1
BMVXCPBXGZKUPN-UHFFFAOYSA-N	hexylamine	1
BNWJOHGLIBDBOB-UHFFFAOYSA-N	myristicin	1
BOTWFXYSPFMFNR-PYDDKJGSSA-N	phytol	1
BQOFWKZOCNGFEC-BDAKNGLRSA-N	(1s)-(+)-3-carene	1
BSYNRYMUTXBXSQ-UHFFFAOYSA-N	acetylsalicylic acid	1
BTANRVKWQNVYAZ-BYPYZUCNSA-N	(+)-2-Butanol	1
BTANRVKWQNVYAZ-UHFFFAOYSA-N	2-Butanol	1
BTKXLQSCEOHKTF-SREVYHEPSA-N	Z11-hexdecenyl acetate	1
BTSIZIIPFNVMHF-ARJAWSKDSA-N	cis-2-penten-1-ol	1
BWVZAZPLUTUBKD-UHFFFAOYSA-N	3-[5,5,6-trimethylbicyclo[2.2.1]hept-2-yl]cyclohexan-1-ol	1
BXWNKGSJHAJOGX-UHFFFAOYSA-N	1-hexadecanol	1
CBJPZHSWLMJQRI-UHFFFAOYSA-N	furfuryl disulfide	1
CBOQJANXLMLOSS-UHFFFAOYSA-N	ethyl vanillin	1
CCCMONHAUSKTEQ-UHFFFAOYSA-N	1-octadecene	1
CDOSHBSSFJOMGT-SNVBAGLBSA-N	s-(+)linalool	1
CERQOIWHTDAKMF-UHFFFAOYSA-N	methacrylic acid	1
CFAKWWQIUFSQFU-UHFFFAOYSA-N	2-hydroxy-3-methyl-2-cyclopenten-1-one	1
CHLICZRVGGXEOD-UHFFFAOYSA-N	4-methyl anisole	1
CKLJMWTZIZZHCS-UHFFFAOYSA-N	l-aspartic acid	1
CLUWOWRTHNNBBU-UHFFFAOYSA-N	methional	1
CMPVUVUNJQERIT-UHFFFAOYSA-N	2-Isobutylthiazole	1
CPLJMYOQYRCCBY-UHFFFAOYSA-N	2-n-propylfuran	1
CRDAMVZIKSXKFV-UHFFFAOYSA-N	Farnesol	1
CRPUJAZIXJMDBK-BDAKNGLRSA-N	(-)-camphene	1
CRPUJAZIXJMDBK-DTWKUNHWSA-N	(+)-camphene	1
CTQNGGLPUBDAKN-UHFFFAOYSA-N	o-xylene	1
CXWXQJXEFPUFDZ-UHFFFAOYSA-N	1,2,3,4-tetrahydronaphthalene	1
CZASMUMJSKOHFJ-UHFFFAOYSA-N	1-bromoeicosane	1
DJOCFLQKCMWABC-UHFFFAOYSA-N	2-pentyl butyrate	1
DLVYTANECMRFGX-UHFFFAOYSA-N	4-hydroxy-5-methyl-3-furanone	1
DSSYKIVIOFKYAU-OMNKOJBGSA-N	(-)-camphor	1
DSSYKIVIOFKYAU-UHFFFAOYSA-N	camphor (+ -)	1
DTGKSKDOIYIVQL-XKSSXDPKSA-N	isoborneol	1
DTGKSKDOIYIVQL-ZCUBBSJVSA-N	(+)-borneol	1
DTGXDHGNUDOWJS-UHFFFAOYSA-N	4-pentenyl butanoate	1
DYLPEFGBWGEFBB-OSFYFWSMSA-N	(+)-ß-cedrene	1
DZAUWHJDUNRCTF-UHFFFAOYSA-N	3,4-dihydroxyhydrocinnamic acid	1
FDPIMTJIUBPUKL-UHFFFAOYSA-N	3-Pentanone	1
FGPPDYNPZTUNIU-UHFFFAOYSA-N	pentyl valerate	1
FHKSXSQHXQEMOK-LURJTMIESA-N	1,2- hexanediol	1
FQTLCLSUCSAZDY-SDNWHVSQSA-N	trans-nerolidol	1
FUDNBFMOXDUIIE-JTQLQIEISA-N	(-)-ß-citronellene	1
FZWLAAWBMGSTSO-UHFFFAOYSA-N	Thiazole	1
GEWDNTWNSAZUDX-WQMVXFAESA-N	methyl jasmonate	1
GHVNFZFCNZKVNT-UHFFFAOYSA-N	decanoic acid	1
GJQIMXVRFNLMTB-UHFFFAOYSA-N	nonyl acetate	1
GJWSUKYXUMVMGX-UHFFFAOYSA-N	citronellic acid	1
GLDOVTGHNKAZLK-UHFFFAOYSA-N	1-octadecanol	1
GRHQDJDRGZFIPO-UHFFFAOYSA-N	4-bromobutyric acid	1
GWHJZXXIDMPWGX-UHFFFAOYSA-N	1,2,4-trimethylbenzene	1
GXANMBISFKBPEX-ARJAWSKDSA-N	cis-3-hexanal	1
GYCKQBWUSACYIF-UHFFFAOYSA-N	ethyl salicylate	1
HFJRKMMYBMWEAD-UHFFFAOYSA-N	dodecyl aldehyde	1
HFLGBNBLMBSXEM-UHFFFAOYSA-N	4-ethylcatechol	1
HHVIBTZHLRERCL-UHFFFAOYSA-N	dimethyl sulfone	1
HITOXZPZGPXYHY-UJURSFKZSA-N	levoglucosenone	1
HLZKNKRTKFSKGZ-UHFFFAOYSA-N	1-tetradecanol	1
HNJBEVLQSNELDL-UHFFFAOYSA-N	2-pyrrolidinone	1
HTSABYAWKQAHBT-UHFFFAOYSA-N	3-methylcyclohexanol, mixture of cis and trans	1
HYFLWBNQFMXCPA-UHFFFAOYSA-N	2-ethyltoluene	1
IAIHUHQCLTYTSF-OYNCUSHFSA-N	(1R)-endo-(+)-fenchyl alcohol	1
IEPWIPZLLIOZLU-ONEGZZNKSA-N	cis-3-hexenyl salicylate	1
IGIDLTISMCAULB-UHFFFAOYSA-N	3-methylpentanoic acid	1
IHPKGUQCSIINRJ-NTMALXAHSA-N	z-(ß)-ocimene	1
IOJUPLGTWVMSFF-UHFFFAOYSA-N	benzothiazole	1
IRAQOCYXUMOFCW-OSFYFWSMSA-N	(-)-alpha-cedrene	1
IVSZLXZYQVIEFR-UHFFFAOYSA-N	m-xylene	1
IWTBVKIGCDZRPL-UHFFFAOYSA-N	3-methyl-1-pentanol	1
JGMYDQCXGIMHLL-WAYWQWQTSA-N	z-11-hexadecenoic acid	1
JJYWRQLLQAKNAD-SNAWJCMRSA-M	2-methyl-2-pentenoic acid	1
JKOTZBXSNOGCIF-UHFFFAOYSA-N	1-bromopentadecane	1
JNIWCVWKECYDSV-UHFFFAOYSA-N	Furfuryl Octanoate	1
JNWQKXUWZWKUAY-BAGHCZIJSA-N	cis-3-hexenyl tiglate	1
JOZKFWLRHCDGJA-UHFFFAOYSA-N	Citronellyl acetate	1
JRLPEMVDPFPYPJ-UHFFFAOYSA-N	4-ethyl toluene	1
JVTAAEKCZFNVCJ-REOHCLBHSA-N	l-(+)-lactic acid	1
KBPUBCVJHFXPOC-UHFFFAOYSA-N	ethyl protocatechuate	1
KDSNLYIMUZNERS-UHFFFAOYSA-N	isobutylamine	1
KEBDNKNVCHQIJU-UHFFFAOYSA-N	isobutyl isovalerate	1
KFZMGEQAYNKOFK-UHFFFAOYSA-N	isopropyl alcohol	1
KFZXVMNBUMVKLN-UHFFFAOYSA-N	4-chloro-2-isopropyl-5-methylphenol	1
KGWPHCDTOLQQEP-UHFFFAOYSA-N	7-methylindole	1
KITGYVIOYOCIIE-PTFSRLPTSA-N	cis-2-hexenyl crotonate	1
KMPQYAYAQWNLME-UHFFFAOYSA-N	undecanal	1
KRIOVPPHQSLHCZ-UHFFFAOYSA-N	propiophenone	1
KSEBMYQBYZTDHS-HWKANZROSA-N	ferulic acid	1
KSXTZYRIJKDCEA-UHFFFAOYSA-N	3,7-dimethyl-1-octene	1
KWKAKUADMBZCLK-UHFFFAOYSA-N	1-octene	1
KYWIYKKSMDLRDC-UHFFFAOYSA-N	2-undecanone	1
LCOSCMLXPAQCLQ-UHFFFAOYSA-N	beta-himachalene	1
LGYNIFWIKSEESD-UHFFFAOYSA-N	2-ethyl hexanal	1
LLHKCFNBLRBOGN-UHFFFAOYSA-N	propylene glycol monomethyl ether acetate	1
LPWKTEHEFDVAQS-VOTSOKGWSA-N	trans-2-hexenyl propionate	1
LQAVWYMTUMSFBE-UHFFFAOYSA-N	4-Penten-1-ol	1
LQKRYVGRPXFFAV-UHFFFAOYSA-N	ethyl 3-methyl-3-phenylglycidate	1
LUYISICIYVKBTA-UHFFFAOYSA-N	6-methylquinoline	1
LVSQXDHWDCMMRJ-UHFFFAOYSA-N	4-hydroxy-2-butanone	1
LZCLXQDLBQLTDK-BYPYZUCNSA-N	(-)-ethyl l-lactate	1
MCSINKKTEDDPNK-UHFFFAOYSA-N	propyl propionate	1
MFKRHJVUCZRDTF-UHFFFAOYSA-N	3-methoxy-3-methyl-1-butanol	1
MGSRCZKZVOBKFT-UHFFFAOYSA-N	thymol	1
MIJDSYMOBYNHOT-UHFFFAOYSA-N	2-(ethylamino)ethanol	1
MNDIARAMWBIKFW-UHFFFAOYSA-N	1-bromohexane	1
MOMFXATYAINJML-UHFFFAOYSA-N	2-Acetylthiazole	1
MPKOLWUWOADQCX-BMRADRMJSA-N	alpha-pentylcinnamaldehyde diethyl acetal	1
MSXVEPNJUHWQHW-UHFFFAOYSA-N	2-methyl-2-butanol	1
MUXOBHXGJLMRAB-UHFFFAOYSA-N	dimethyl succinate	1
MVPPADPHJFYWMZ-UHFFFAOYSA-N	Chlorobenzene	1
MYMSJFSOOQERIO-UHFFFAOYSA-N	1-bromodecane	1
NDVASEGYNIMXJL-UHFFFAOYSA-N	(+/-)-sabinene	1
NDVWOBYBJYUSMF-UHFFFAOYSA-N	2-Methylcyclohexanol	1
NGSWKAQJJWESNS-ZZXKWVIFSA-N	p-coumaric acid	1
NHKIYYMFGJBOTK-UHFFFAOYSA-N	2,5-dimethyl-2,5-dihydroxy-1,4-dithiane	1
NIXOWILDQLNWCW-UHFFFAOYSA-N	acrylic acid	1
NKCQEIXYLHACJC-UHFFFAOYSA-N	6-isopropylquinoline	1
NKDDWNXOKDWJAK-UHFFFAOYSA-N	Dimethoxymethane	1
NLZUEZXRPGMBCV-UHFFFAOYSA-N	butylhydroxytoluene	1
NMHMNPHRMNGLLB-UHFFFAOYSA-N	3-(4-hydroxyphenyl)propionic acid	1
NNWHUJCUHAELCL-SNAWJCMRSA-N	methyl isoeugenol	1
NSSALFVIQPAIQK-BQYQJAHWSA-N	cis-2-nonen-1-ol	1
NUPSHWCALHZGOV-UHFFFAOYSA-N	decyl acetate	1
NVEQFIOZRFFVFW-RGCMKSIDSA-N	(-)-caryophyllene oxide	1
NVRVNSHHLPQGCU-UHFFFAOYSA-M	6-bromohexanoic acid	1
NWZQCEQAPBRMFX-UHFFFAOYSA-N	isobutyl-2-methylbutyrate	1
OCWLYWIFNDCWRZ-UHFFFAOYSA-N	methyl 2-methylbutyrate	1
ODLMAHJVESYWTB-UHFFFAOYSA-N	propyl benzene	1
ODNRTOSCFYDTKF-UHFFFAOYSA-N	1,3,5-trimethylcyclohexane	1
OFBQJSOFQDEBGM-UHFFFAOYSA-N	Pentane	1
OHEFFKYYKJVVOX-MRVPVSSYSA-N	(r)-6-methyl-5-hepten-2-ol	1
OHEFFKYYKJVVOX-QMMMGPOBSA-N	(s)-6-methyl-5-hepten-2-ol	1
OHEFFKYYKJVVOX-UHFFFAOYSA-N	(±)-6-methyl-5-hepten-2-ol	1
OJVAMHKKJGICOG-UHFFFAOYSA-N	2,5-hexanedione	1
OKJPEAGHQZHRQV-UHFFFAOYSA-N	iodoform	1
ONKNPOPIGWHAQC-UHFFFAOYSA-N	galaxolide	1
OPFTUNCRGUEPRZ-UHFFFAOYSA-N	beta-elemene	1
OSMAJVWUIUORGC-WAYWQWQTSA-N	cis-3-hexenyl isobutyrate	1
OTKQNSSMCDLVQV-UHFFFAOYSA-N	butyl 2-methylbutyrate	1
OUYCCCASQSFEME-UHFFFAOYSA-N	l-tyrosine	1
OVJCOXQJBRJIRJ-OVCLIPMQSA-N	maritima	1
PCGACKLJNBBQGM-VOTSOKGWSA-N	trans-2-hexenyl butyrate	1
PDSNLYSELAIEBU-UHFFFAOYSA-N	(+)-longifolene	1
PHXATPHONSXBIL-JTQLQIEISA-N	undecanoic -gamma-lactone	1
PIICEJLVQHRZGT-UHFFFAOYSA-N	ethylenediamine	1
POAOYUHQDCAZBD-UHFFFAOYSA-N	ethylene glycol butyl ether	1
PPBRXRYQALVLMV-UHFFFAOYSA-N	styrene	1
QAOXMQCWUWZZNC-ONEGZZNKSA-M	4-methyl-2-pentenoic acid	1
QARVLSVVCXYDNA-UHFFFAOYSA-N	bromobenzene	1
QCDWFXQBSFUVSP-UHFFFAOYSA-N	2-phenoxyethanol	1
QIQXTHQIDYTFRH-UHFFFAOYSA-N	stearic acid	1
QJJDNZGPQDGNDX-UHFFFAOYSA-N	dihydro-ß-ionone	1
QJQZRLXDLORINA-UHFFFAOYSA-N	2-cyclohexylethanol	1
QMHIMXFNBOYPND-UHFFFAOYSA-N	4-Methylthiazole	1
QMVPMAAFGQKVCJ-UHFFFAOYSA-N	β-Citronellol	1
QPRQEDXDYOZYLA-UHFFFAOYSA-N	2-methyl-1-butanol	1
QRGSTISKDZCDHV-XCVCLJGOSA-N	Benzyl trans-2-methyl-2-butenoate	1
QUEBYVKXYIKVSO-UHFFFAOYSA-N	3-propyltoluene	1
QYDYPVFESGNLHU-ZHACJKMWSA-N	methyl oleate	1
REFJWTPEDVJJIY-UHFFFAOYSA-N	quercetin	1
REIUXOLGHVXAEO-UHFFFAOYSA-N	1-pentadecanol	1
RFFLAFLAYFXFSW-UHFFFAOYSA-N	o-dichlorobenzene	1
RGACQXBDYBCJCY-ALCCZGGFSA-N	cis-3-hexenyl hexanoate	1
RGFNRWTWDWVHDD-UHFFFAOYSA-N	isobutyl butyrate	1
RHVMNRHQWXIJIS-WAYWQWQTSA-N	z-11-hexadecen-1-ol	1
RKFAZBXYICVSKP-WAYWQWQTSA-N	2,4,5-trimethoxy-1-propenylbenzene	1
RRHGJUQNOFWUDK-UHFFFAOYSA-N	isoprene	1
RSJKGSCJYJTIGS-UHFFFAOYSA-N	undecane	1
RTZZCYNQPHTPPL-UHFFFAOYSA-N	3-nitrophenol	1
RWCCWEUUXYIKHB-UHFFFAOYSA-N	benzophenone	1
RWGFKTVRMDUZSP-UHFFFAOYSA-N	cumene	1
RXGUIWHIADMCFC-UHFFFAOYSA-N	isobutyl isobutyrate	1
RZKSECIXORKHQS-UHFFFAOYSA-N	(+)-3-Heptanol	1
SECXISVLQFMRJM-UHFFFAOYSA-N	1-methyl-2-pyrrolidinone	1
SGVYKUFIHHTIFL-UHFFFAOYSA-N	2-methylnonane	1
SJWFXCIHNDVPSH-UHFFFAOYSA-N	2-octanol	1
SNICXCGAKADSCV-JTQLQIEISA-N	l-(-)-nicotine	1
SNRUBQQJIBEYMU-UHFFFAOYSA-N	dodecane	1
SPEUIVXLLWOEMJ-UHFFFAOYSA-N	1,1-Dimethoxyethane	1
SQIPCPAQCPMJFW-UHFFFAOYSA-N	3-methyl-2-butenyl butanoate	1
STJIISDMSMJQQK-UHFFFAOYSA-N	furan-3-methanol	1
SWXVUIWOUIDPGS-UHFFFAOYSA-N	4-hydroxy-4-methyl-2-pentanone	1
SXOZDDAFVJANJP-UHFFFAOYSA-N	cyclodecanone	1
SYBYTAAJFKOIEJ-UHFFFAOYSA-N	3-methyl-2-butanone	1
TWLLPUMZVVGILS-UHFFFAOYSA-N	ethyl 2-aminobenzoate	1
UBLAMKHIFZBBSS-UHFFFAOYSA-N	isoamyl valerate	1
UEEJHVSXFDXPFK-UHFFFAOYSA-N	2-dimethylaminoethanol	1
UERBALMVMSYIMI-UHFFFAOYSA-N	Xylenes	1
UFWIBTONFRDIAS-UHFFFAOYSA-N	naphthalene	1
UIERETOOQGIECD-ARJAWSKDSA-N	2-methyl-cis-2-butenoic acid	1
UIERETOOQGIECD-ONEGZZNKSA-N	trans-2-methyl-2-butenoic acid	1
UIUWNILCHFBLEQ-NSCUHMNNSA-N	3-pentenoic acid	1
UJPPXNXOEVDSRW-UHFFFAOYSA-N	isopropyl laurate	1
UNNGUFMVYQJGTD-UHFFFAOYSA-N	2-ethylbutyraldehyde	1
UPMAOXLCTXPPAG-UHFFFAOYSA-N	decahydro-2-naphthol	1
UPOSSYJVWXLPTA-UHFFFAOYSA-N	cycloundecanone	1
UQPLEMTXCSYMEK-VQHVLOKHSA-N	n-caproic acid trans-2-hexen-1-yl ester	1
URAYPUMNDPQOKB-UHFFFAOYSA-N	triacetin	1
URLKBWYHVLBVBO-UHFFFAOYSA-N	p-xylene anhydrous	1
UVZICZIVKIMRNE-UHFFFAOYSA-N	2,2'-thiodiacetic acid	1
UWSONZCNXUSTKW-UHFFFAOYSA-N	4,5-Dimethylthiazole	1
UXUPPWPIGVTVQI-UHFFFAOYSA-N	isobutyl hexanoate	1
VAMXMNNIEUEQDV-UHFFFAOYSA-N	methyl 2-aminobenzoate	1
VBCIOOKAKHGVMI-UHFFFAOYSA-N	5-methyl-5-hexen-2-one	1
VDHRTASWKDTLER-ARJAWSKDSA-N	cis-5-octen-1-ol	1
VFPFQHQNJCMNBZ-UHFFFAOYSA-N	ethyl gallate	1
VGIRHYHLQKDEPP-UHFFFAOYSA-N	isoamyl 2-methylbutyrate	1
VHOMAPWVLKRQAZ-UHFFFAOYSA-N	benzyl propionate	1
VLXDPFLIRFYIME-ZPWMNFGTSA-N	(-)-a-copaene	1
VSMOENVRRABVKN-QMMMGPOBSA-N	(r)-(-)-1-octen-3-ol	1
VTWDKFNVVLAELH-UHFFFAOYSA-N	p-toluquinone	1
VZRGLBPRLIGTPE-UHFFFAOYSA-N	Furfuryl heptanoate	1
WBRFDUJXCLCKPX-UHFFFAOYSA-N	2,3-dimethyl heptane	1
WDAXFOBOLVPGLV-UHFFFAOYSA-N	Ethyl Isobutyrate	1
WERYXYBDKMZEQL-UHFFFAOYSA-N	1,4-butanediol	1
WGGJTPQHVFOGPN-PLNGDYQASA-N	cis-2-penten-1-yl acetate	1
WGQKYBSKWIADBV-UHFFFAOYSA-N	Benzylamine	1
WGYKZJWCGVVSQN-UHFFFAOYSA-N	propylamine	1
WHWDWIHXSPCOKZ-UHFFFAOYSA-N	6,10,14-trimethyl-2-pentadecanone	1
WLAMNBDJUVNPJU-UHFFFAOYSA-N	(±)-2-methylbutyric acid	1
WNXNUPJZWYOKMW-UHFFFAOYSA-N	5-bromovaleric acid	1
WPYMKLBDIGXBTP-UHFFFAOYSA-N	benzoic acid	1
WQOXQRCZOLPYPM-UHFFFAOYSA-N	dimethyl disulfide	1
WTEVQBCEXWBHNA-UHFFFAOYSA-N	Citral	1
WTOYNNBCKUYIKC-JMSVASOKSA-N	(+)-nootkatone	1
WTTJVINHCBCLGX-NQLNTKRDSA-N	methyl linoleate	1
WXCMHFPAUCOJIG-UHFFFAOYSA-N	musk ketone	1
XEAIHUDTEINXFG-SNAWJCMRSA-N	methyl trans-3-hexenoate	1
XEFAJZOBODPHBG-UHFFFAOYSA-N	phenoxyethanol	1
XFRVVPUIAFSTFO-UHFFFAOYSA-N	1-tridecanol	1
XINCECQTMHSORG-UHFFFAOYSA-N	isoamyl isovalerate	1
XJHQVZQZUGLZLS-ARJAWSKDSA-N	cis-3-hexenyl formate	1
XOAAWQZATWQOTB-UHFFFAOYSA-N	taurine	1
XPFTVTFOOTVHIA-FNORWQNLSA-N	hexenyl-cis-3-valerate	1
XUGNVMKQXJXZCD-UHFFFAOYSA-N	isopropyl palmitate	1
XUWHAWMETYGRKB-UHFFFAOYSA-N	d-valerolactam	1
XXHDAWYDNSXJQM-ARJAWSKDSA-N	3-hexenoic acid, (3z)-	1
XXHDAWYDNSXJQM-ONEGZZNKSA-N	trans-3-hexenoic acid	1
YCCILVSKPBXVIP-UHFFFAOYSA-N	tyrosol	1
YFTHZRPMJXBUME-UHFFFAOYSA-N	tripropylamine	1
YHRUHBBTQZKMEX-YFVJMOTDSA-N	farnesal	1
YMWUJEATGCHHMB-UHFFFAOYSA-N	dichloromethane	1
YPKBCLZFIYBSHK-UHFFFAOYSA-N	5-methylindole	1
YPWFNLSXQIGJCK-UHFFFAOYSA-N	7-Oxabicyclo[2.2.1]-heptane	1
YRKCREAYFQTBPV-UHFFFAOYSA-N	acetylacetone	1
YSHXMTRDMDYAEC-UHFFFAOYSA-N	6-pentadecanone	1
YSJHMPXIMIOXGU-UHFFFAOYSA-N	vertenol-iso-butyrate	1
YTHRBOFHFYZBRJ-UHFFFAOYSA-N	(-)-carvenyl acetate	1
YWHLKYXPLRWGSE-UHFFFAOYSA-N	dimethyl trisulfide	1
YYPNJNDODFVZLE-UHFFFAOYSA-N	3,3-dimethylacrylic acid	1
ZAJNGDIORYACQU-UHFFFAOYSA-N	2-Decanone	1
ZCFOBLITZWHNNC-VOTSOKGWSA-N	trans-3-octen-2-one	1
ZCHOPXVYTWUHDS-WAYWQWQTSA-N	cis-3-hexenyl butyrate	1
ZJLMKPKYJBQJNH-UHFFFAOYSA-N	1,3-Propanedithiol	1
ZLCSFXXPPANWQY-UHFFFAOYSA-N	3-ethyl toluene	1
ZMANZCXQSJIPKH-UHFFFAOYSA-N	triethylamine	1
ZOJBYZNEUISWFT-UHFFFAOYSA-N	allyl isothiocyanate	1
ZQPPMHVWECSIRJ-MDZDMXLPSA-N	oleic acid	1

Table S5. Related to Figure 2. Data from calcium imaging. Response values are mean deltaF/F values ± SEM. Corresponding solvent responses were subtracted from all responses. Values in parentheses indicate n.

odorant	InChIKey	class	Or10a	Or42b	Or47b	Or56a	Or69a
acetic acid	QTBSBXVTEAMEQO-UHFFFAOYSA-N	acid	-1.05 ± 0.45 (6)	-0.32 ± 0.08 (5)	-0.9 ± 0.19 (10)	-3.53 ± 0.39 (12)	0.14 ± 0.12 (7)
butyric acid	FERIUCNNQQJTOY-UHFFFAOYSA-N	acid	1.72 ± 0.24 (4)	-0.2 ± 0.06 (5)	NA	-0.56 ± 0.32 (8)	0.84 ± 0.2 (7)
heptanoic acid	MNWFXJYAOYHMED-UHFFFAOYSA-N	acid	-0.05 ± 0.15 (9)	-0.06 ± 0.06 (5)	0.04 ± 0.07 (11)	-0.28 ± 0.13 (9)	-0.26 ± 0.08 (7)
hexanoic acid	FUZZWVXGSFPDMH-UHFFFAOYSA-N	acid	-0.24 ± 0.21 (10)	-0.06 ± 0.11 (5)	0.09 ± 0.05 (11)	-0.67 ± 0.2 (9)	-0.38 ± 0.07 (7)
isobutyric acid	KQNPFQTWMSNSAP-UHFFFAOYSA-N	acid	0.43 ± 0.16 (10)	-0.2 ± 0.07 (5)	0.38 ± 0.19 (11)	-1.48 ± 0.54 (9)	0.4 ± 0.14 (7)
iso-pentanoic acid	GWYFCOCPABKNJV-UHFFFAOYSA-N	acid	NA	NA	NA	NA	0.48 ± 0.03 (125)
nonanoic acid	FBUKVWPVBMHYJY-UHFFFAOYSA-N	acid	-0.12 ± 0.11 (10)	-0.05 ± 0.07 (5)	0.4 ± 0.22 (9)	-0.01 ± 0.09 (9)	0.02 ± 0.08 (7)
octanoic acid	WWZKQHOCKIZLMA-UHFFFAOYSA-N	acid	-0.16 ± 0.14 (9)	-0.04 ± 0.1 (5)	0.21 ± 0.08 (9)	-0.02 ± 0.14 (9)	-0.33 ± 0.11 (7)
pentanoic acid	NQPDZGIKBAWPEJ-UHFFFAOYSA-N	acid	0.12 ± 0.16 (10)	-0.03 ± 0.06 (5)	0.31 ± 0.08 (11)	-0.78 ± 0.33 (8)	1 ± 0.27 (7)
propanoic acid	XBDQKXXYIPTUBI-UHFFFAOYSA-N	acid	1.1 ± 0.29 (8)	0.09 ± 0.07 (5)	0.79 ± 0.17 (8)	-1.23 ± 0.43 (8)	1 ± 0.27 (7)
1-hexen-3-ol	BVOSSZSHBZQJOI-UHFFFAOYSA-N	alcohol	0.19 ± 0.17 (8)	0.03 ± 0.21 (5)	-0.06 ± 0.05 (8)	1.41 ± 0.3 (7)	0.66 ± 0.16 (7)
2,3-butanediol	OWBTYPJTUOEWEK-UHFFFAOYSA-N	alcohol	0.19 ± 0.18 (10)	-0.4 ± 0.08 (5)	0.09 ± 0.08 (11)	0.12 ± 0.23 (8)	0.16 ± 0.16 (7)
2-heptanol	CETWDUZRCINIHU-UHFFFAOYSA-N	alcohol	-0.02 ± 0.09 (10)	-0.41 ± 0.05 (5)	-0.18 ± 0.1 (8)	1.81 ± 0.19 (11)	1.48 ± 0.31 (7)
2-hexanol	QNVRIHYSUZMSGM-UHFFFAOYSA-N	alcohol	0.35 ± 0.11 (10)	1.5 ± 0.17 (5)	-0.45 ± 0.05 (8)	2.28 ± 0.04 (295)	0.79 ± 0.23 (7)
3-methyl-butanol	PHTQWCKDNZKARW-UHFFFAOYSA-N	alcohol	0.52 ± 0.16 (8)	-0.26 ± 0.06 (5)	-0.14 ± 0.04 (8)	0.38 ± 0.21 (8)	0.38 ± 0.17 (7)
3-octanol	NMRPBPVERJPACX-UHFFFAOYSA-N	alcohol	0.52 ± 0.15 (7)	-0.47 ± 0.04 (5)	-0.11 ± 0.09 (8)	0.38 ± 0.14 (9)	1.68 ± 0.47 (7)
4-methylcyclohexanol	MQWCXKGKQLNYQG-UHFFFAOYSA-N	alcohol	0.53 ± 0.16 (9)	-0.18 ± 0.05 (5)	0.01 ± 0.08 (8)	1.87 ± 0.34 (9)	1.33 ± 0.27 (7)
4-octanol	WOFPPJOZXUTRAU-UHFFFAOYSA-N	alcohol	0.36 ± 0.15 (9)	-0.38 ± 0.07 (5)	-0.1 ± 0.08 (8)	0.73 ± 0.12 (9)	1.38 ± 0.26 (7)
decanol	MWKFXSUHUHTGQN-UHFFFAOYSA-N	alcohol	-0.15 ± 0.12 (10)	-0.07 ± 0.05 (5)	0.34 ± 0.07 (9)	-0.13 ± 0.12 (9)	1.27 ± 0.25 (7)
geosmin	JLPUXFOGCDVKGO-GRYCIOLGSA-N	alcohol	NA	NA	NA	17.59 ± 4.41 (4)	NA
hexanol	ZSIAUFGUXNUGDI-UHFFFAOYSA-N	alcohol	NA	NA	-0.06 ± 0.06 (9)	NA	NA
Z3-hexenol	UFLHIIWVXFIJGU-ARJAWSKDSA-N	alcohol	0.1 ± 0.12 (8)	0.27 ± 0.25 (5)	-0.17 ± 0.06 (8)	0.97 ± 0.22 (8)	0.82 ± 0.22 (7)
acetaldehyde	IKHGUXGNUITLKF-UHFFFAOYSA-N	aldehyde	NA	-0.29 ± 0.1 (9)	NA	NA	NA
butanal	ZTQSAGDEMFDKMZ-UHFFFAOYSA-N	aldehyde	-0.25 ± 0.17 (9)	-0.4 ± 0.14 (9)	-0.24 ± 0.11 (8)	0.44 ± 0.17 (9)	0.35 ± 0.06 (6)
decanal	KSMVZQYAVGTKIV-UHFFFAOYSA-N	aldehyde	0.2 ± 0.1 (10)	-0.08 ± 0.05 (9)	-0.13 ± 0.07 (8)	0.01 ± 0.12 (10)	0.29 ± 0.09 (6)
E2-hexenal	MBDOYVRWFFCFHM-SNAWJCMRSA-N	aldehyde	0.03 ± 0.22 (8)	0.12 ± 0.17 (9)	0.39 ± 0.24 (10)	0.35 ± 0.13 (8)	0.16 ± 0.13 (7)
heptanal	FXHGMKSSBGDXIY-UHFFFAOYSA-N	aldehyde	0.13 ± 0.17 (8)	-0.48 ± 0.12 (9)	-0.13 ± 0.06 (8)	0.04 ± 0.13 (8)	-0.09 ± 0.13 (6)
hexanal	JARKCYVAAOWBJS-UHFFFAOYSA-N	aldehyde	0.01 ± 0.16 (9)	-0.46 ± 0.11 (9)	-0.36 ± 0.08 (8)	0.17 ± 0.19 (9)	-0.16 ± 0.12 (6)
nonanal	GYHFUZHODSMOHU-UHFFFAOYSA-N	aldehyde	0.15 ± 0.09 (11)	-0.13 ± 0.06 (9)	-0.42 ± 0.06 (11)	-0.18 ± 0.11 (13)	0.46 ± 0.1 (6)
octanal	NUJGJRNETVAIRJ-UHFFFAOYSA-N	aldehyde	0.17 ± 0.11 (10)	-0.11 ± 0.08 (9)	-0.39 ± 0.1 (8)	-0.28 ± 0.18 (10)	0.21 ± 0.14 (6)
pentanal	HGBOYTHUEUWSSQ-UHFFFAOYSA-N	aldehyde	-0.06 ± 0.13 (8)	-0.29 ± 0.1 (9)	-0.15 ± 0.09 (8)	0.18 ± 0.15 (8)	0.07 ± 0.13 (6)
propanal	NBBJYMSMWIIQGU-UHFFFAOYSA-N	aldehyde	-1.01 ± 0.41 (8)	-0.46 ± 0.19 (9)	-0.74 ± 0.24 (9)	0.84 ± 0.19 (9)	0.58 ± 0.07 (6)
heptane	IMNFDUFMRHMDMM-UHFFFAOYSA-N	alkane	0.39 ± 0.15 (8)	0.01 ± 0.07 (5)	0.03 ± 0.03 (8)	0.26 ± 0.08 (8)	0.02 ± 0.08 (7)
nonane	BKIMMITUMNQMOS-UHFFFAOYSA-N	alkane	0.31 ± 0.13 (8)	0 ± 0.06 (4)	0.09 ± 0.04 (8)	-0.02 ± 0.13 (7)	-0.1 ± 0.06 (7)
octane	TVMXDCGIABBOFY-UHFFFAOYSA-N	alkane	0.53 ± 0.15 (7)	0.21 ± 0.04 (5)	0.06 ± 0.03 (8)	-0.03 ± 0.16 (8)	0.04 ± 0.1 (7)
2-ethylphenol	IXQGCWUGDFDQMF-UHFFFAOYSA-N	aromatic	0.31 ± 0.12 (10)	-0.6 ± 0.13 (8)	-0.44 ± 0.04 (8)	-0.54 ± 0.13 (10)	0.42 ± 0.11 (7)
2-propylphenol	LCHYEKKJCUJAKN-UHFFFAOYSA-N	aromatic	-0.57 ± 0.06 (10)	-0.44 ± 0.11 (8)	-0.21 ± 0.09 (11)	-0.75 ± 0.16 (12)	0.45 ± 0.06 (7)
4-allyl-1,2-dimethoxybenzene	ZYEMGPIYFIJGTP-UHFFFAOYSA-N	aromatic	0.53 ± 0.12 (11)	-0.28 ± 0.1 (8)	0.11 ± 0.05 (11)	0.01 ± 0.13 (12)	0.43 ± 0.09 (7)
4hydroxy-3-methoxybenzaldehyde	RUVINXPYWBROJD-ONEGZZNKSA-N	aromatic	0.26 ± 0.09 (10)	0.03 ± 0.1 (9)	0.19 ± 0.09 (11)	-0.04 ± 0.11 (12)	0.34 ± 0.07 (6)
4-isopropylbenzaldehyde	WTWBUQJHJGUZCY-UHFFFAOYSA-N	aromatic	0.21 ± 0.1 (6)	-0.15 ± 0.06 (9)	NA	-0.45 ± 0.12 (12)	0.37 ± 0.07 (6)
4-methoxybenzaldehyde	ZRSNZINYAWTAHE-UHFFFAOYSA-N	aromatic	0.61 ± 0.1 (11)	-0.17 ± 0.08 (9)	0.67 ± 0.11 (10)	0.01 ± 0.21 (12)	1.02 ± 0.14 (6)
4-methoxybenzene	RDOXTESZEPMUJZ-UHFFFAOYSA-N	aromatic	0.85 ± 0.16 (10)	-0.26 ± 0.1 (9)	0.01 ± 0.05 (8)	-0.37 ± 0.19 (10)	0.18 ± 0.06 (6)
4-methylphenol	IWDCLRJOBJJRNH-UHFFFAOYSA-N	aromatic	0.94 ± 0.19 (8)	-0.52 ± 0.13 (8)	0.16 ± 0.1 (8)	-0.21 ± 0.2 (9)	1.17 ± 0.14 (7)
benzaldehyde	HUMNYLRZRPPJDN-UHFFFAOYSA-N	aromatic	0.45 ± 0.27 (8)	-0.36 ± 0.12 (9)	-1.36 ± 0.4 (8)	-0.67 ± 0.09 (8)	-0.26 ± 0.11 (6)
benzonitrile	JFDZBHWFFUWGJE-UHFFFAOYSA-N	aromatic	1.1 ± 0.18 (8)	-0.54 ± 0.14 (8)	0.06 ± 0.07 (8)	-0.35 ± 0.09 (8)	0.93 ± 0.07 (7)
benzyl alcohol	WVDDGKGOMKODPV-UHFFFAOYSA-N	aromatic	0.68 ± 0.17 (8)	-0.11 ± 0.06 (9)	-0.29 ± 0.09 (8)	-0.21 ± 0.15 (9)	0.17 ± 0.06 (6)
ethyl benzoate	MTZQAGJQAFMTAQ-UHFFFAOYSA-N	aromatic	2.38 ± 0.31 (10)	-0.5 ± 0.2 (7)	-0.06 ± 0.08 (8)	0.45 ± 0.11 (10)	0.75 ± 0.06 (7)
eugenol	RRAFCDWBNXTKKO-UHFFFAOYSA-N	aromatic	0.24 ± 0.11 (11)	-0.1 ± 0.07 (9)	-0.24 ± 0.09 (11)	0.62 ± 0.09 (12)	0.87 ± 0.13 (6)
iso-eugenol	BJIOGJUNALELMI-ONEGZZNKSA-N	aromatic	-0.12 ± 0.18 (11)	-0.16 ± 0.06 (9)	-0.11 ± 0.09 (11)	-0.19 ± 0.11 (12)	0.28 ± 0.09 (6)
methyl jasmonate	GEWDNTWNSAZUDX-SNAWJCMRSA-N	aromatic	0.14 ± 0.09 (11)	-0.34 ± 0.15 (7)	0.06 ± 0.05 (11)	0.13 ± 0.08 (12)	0.15 ± 0.07 (7)
methyl salicylate	OSWPMRLSEDHDFF-UHFFFAOYSA-N	aromatic	2.77 ± 0.43 (10)	-0.35 ± 0.13 (8)	0.31 ± 0.06 (16)	-0.03 ± 0.13 (9)	1.04 ± 0.05 (7)
phenethyl alcohol	WRMNZCZEMHIOCP-UHFFFAOYSA-N	aromatic	0.43 ± 0.18 (9)	-0.15 ± 0.15 (8)	0.02 ± 0.14 (8)	0.69 ± 0.18 (9)	0.8 ± 0.12 (7)
phenylacetaldehyde	DTUQWGWMVIHBKE-UHFFFAOYSA-N	aromatic	0.23 ± 0.11 (6)	-0.31 ± 0.11 (8)	NA	0.12 ± 0.08 (12)	NA
2-methyl butyl acetate	XHIUFYZDQBSEMF-UHFFFAOYSA-N	ester	0.49 ± 0.11 (10)	-0.39 ± 0.08 (5)	-0.18 ± 0.06 (8)	-0.11 ± 0.16 (11)	0.69 ± 0.25 (8)
butyl acetate	DKPFZGUDAPQIHT-UHFFFAOYSA-N	ester	0.64 ± 0.02 (258)	-0.62 ± 0.09 (5)	0.05 ± 0.08 (8)	-0.18 ± 0.15 (7)	0.32 ± 0.13 (7)
E2-hexenyl acetate	HRHOWZHRCRZVCU-WAYWQWQTSA-N	ester	-0.29 ± 0.11 (9)	-0.4 ± 0.08 (5)	-0.39 ± 0.07 (8)	0.34 ± 0.31 (10)	1.11 ± 0.27 (8)
ethyl 2-methyl-2E-butenoate	OAPHLAAOJMTMLY-GQCTYLIASA-N	ester	1.19 ± 0.16 (10)	-0.35 ± 0.16 (5)	0.07 ± 0.1 (8)	0.49 ± 0.19 (10)	0.61 ± 0.09 (8)
ethyl 2-methylbutanoate	HCRBXQFHJMCTLF-UHFFFAOYSA-N	ester	0.22 ± 0.08 (10)	0.98 ± 0.19 (5)	0.01 ± 0.06 (8)	1.43 ± 0.28 (10)	0.46 ± 0.15 (8)
ethyl 3-hydroxyhexanoate	LYRIITRHDCNUHV-UHFFFAOYSA-N	ester	-0.12 ± 0.06 (10)	-0.22 ± 0.03 (5)	0.12 ± 0.07 (11)	0.52 ± 0.16 (10)	1.9 ± 0.43 (8)
ethyl isovalerate	PPXUHEORWJQRHJ-UHFFFAOYSA-N	ester	0.7 ± 0.11 (8)	-0.11 ± 0.17 (5)	0.23 ± 0.07 (8)	0.27 ± 0.14 (8)	0.58 ± 0.12 (8)
ethyl propionate	FKRCODPIKNYEAC-UHFFFAOYSA-N	ester	NA	2.55 ± 0.1 (141)	NA	NA	NA
ethyl (R)-(-)-3-hydroxybutanoate	OMSUIQOIVADKIM-RXMQYKEDSA-N	ester	0.34 ± 0.08 (10)	-0.26 ± 0.23 (8)	0.42 ± 0.05 (11)	1.36 ± 0.23 (10)	0.48 ± 0.09 (8)
ethyl (S)-(+)-3-hydroxybutanoate	OMSUIQOIVADKIM-YFKPBYRVSA-N	ester	0.48 ± 0.1 (10)	2.5 ± 0.38 (4)	-0.04 ± 0.1 (8)	2.29 ± 0.25 (11)	1.57 ± 0.34 (7)
heptyl acetate	ZCZSIDMEHXZRLG-UHFFFAOYSA-N	ester	-0.13 ± 0.08 (10)	0.18 ± 0.12 (5)	-0.01 ± 0.06 (11)	0.04 ± 0.08 (9)	0.67 ± 0.19 (7)
hexyl acetate	AOGQPLXWSUTHQB-UHFFFAOYSA-N	ester	-0.16 ± 0.07 (9)	-0.07 ± 0.19 (5)	0.05 ± 0.14 (11)	-0.39 ± 0.29 (9)	0.92 ± 0.24 (8)
hexyl butyrate	XAPCMTMQBXLDBB-UHFFFAOYSA-N	ester	-0.02 ± 0.1 (9)	-0.12 ± 0.05 (5)	0.31 ± 0.09 (11)	0.05 ± 0.11 (9)	0.62 ± 0.14 (8)
hexyl methanoate	OUGPMNMLWKSBRI-UHFFFAOYSA-N	ester	0.44 ± 0.14 (10)	-0.29 ± 0.07 (5)	-0.1 ± 0.04 (8)	-0.26 ± 0.21 (10)	0.76 ± 0.13 (8)
isoamyl tiglate	ZARFDQHJMNVNLE-WEVVVXLNSA-N	ester	-0.14 ± 0.07 (10)	0.34 ± 0.11 (5)	0.31 ± 0.04 (11)	-0.07 ± 0.16 (10)	0.88 ± 0.1 (8)
isopropyl butyrate	FFOPEPMHKILNIT-UHFFFAOYSA-N	ester	0.13 ± 0.14 (8)	0.61 ± 0.19 (5)	0.03 ± 0.06 (8)	0.23 ± 0.18 (8)	0.6 ± 0.28 (8)
isopropyl tiglate	VUPBIVVRPJDWNW-FNORWQNLSA-N	ester	-0.08 ± 0.09 (9)	-0.38 ± 0.03 (5)	-0.08 ± 0.04 (8)	0.12 ± 0.14 (10)	0.47 ± 0.1 (8)
methyl 3-hydroxy hexanoate	ACCRBMDJCPPJDX-UHFFFAOYSA-N	ester	0.3 ± 0.11 (9)	-0.32 ± 0.07 (8)	0.08 ± 0.09 (11)	1.29 ± 0.23 (10)	1.38 ± 0.12 (8)
methyl tiglate	YYJWBYNQJLBIGS-PLNGDYQASA-N	ester	1.2 ± 0.16 (10)	-0.4 ± 0.04 (5)	0.47 ± 0.08 (8)	-0.07 ± 0.18 (10)	0.72 ± 0.11 (8)
octyl acetate	YLYBTZIQSIBWLI-UHFFFAOYSA-N	ester	0.04 ± 0.11 (4)	0.16 ± 0.06 (5)	NA	0.1 ± 0.1 (9)	0.6 ± 0.11 (8)
Z3-hexenyl acetate	NPFVOOAXDOBMCE-PLNGDYQASA-N	ester	-0.03 ± 0.14 (9)	-0.41 ± 0.07 (5)	-0.19 ± 0.08 (8)	0.21 ± 0.15 (10)	1.16 ± 0.23 (8)
2,3-butanedione	QSJXEFYPDANLFS-UHFFFAOYSA-N	ketone	-0.51 ± 0.23 (9)	1.01 ± 0.23 (3)	-0.17 ± 0.16 (8)	-3.52 ± 0.3 (12)	-0.5 ± 0.08 (6)
2-butanone	ZWEHNKRNPOVVGH-UHFFFAOYSA-N	ketone	-0.24 ± 0.22 (9)	0.78 ± 0.26 (9)	-0.12 ± 0.15 (7)	0.35 ± 0.13 (8)	0.04 ± 0.12 (6)
2-heptanone	CATSNJVOTSVZJV-UHFFFAOYSA-N	ketone	0.2 ± 0.12 (8)	-0.53 ± 0.2 (9)	0.47 ± 0.3 (8)	0.04 ± 0.12 (8)	0.39 ± 0.08 (6)
2-hexanone	QQZOPKMRPOGIEB-UHFFFAOYSA-N	ketone	0.25 ± 0.16 (8)	0.34 ± 0.25 (8)	-0.27 ± 0.03 (8)	0.29 ± 0.21 (8)	0.12 ± 0.1 (6)
2-octanone	ZPVFWPFBNIEHGJ-UHFFFAOYSA-N	ketone	0.61 ± 0.15 (9)	-0.59 ± 0.13 (9)	-0.08 ± 0.1 (8)	0.2 ± 0.09 (9)	0.72 ± 0.15 (6)
3-hexanone	PFCHFHIRKBAQGU-UHFFFAOYSA-N	ketone	-0.13 ± 0.15 (9)	2.9 ± 0.32 (9)	-0.31 ± 0.08 (8)	0.7 ± 0.12 (9)	0.07 ± 0.09 (6)
3-penten-2-one	LABTWGUMFABVFG-ARJAWSKDSA-N	ketone	0.3 ± 0.17 (9)	2.11 ± 0.3 (9)	0.04 ± 0.13 (8)	1.25 ± 0.16 (8)	0.07 ± 0.05 (6)
6-methyl-5-hepten-2-one	UHEPJGULSIKKTP-UHFFFAOYSA-N	ketone	NA	NA	0.58 ± 0.15 (9)	NA	NA
acetone	CSCPPACGZOOCGX-UHFFFAOYSA-N	ketone	-0.22 ± 0.23 (9)	-0.08 ± 0.09 (8)	-0.13 ± 0.08 (8)	0.83 ± 0.09 (8)	0.06 ± 0.06 (6)
cyclohexanone	JHIVVAPYMSGYDF-UHFFFAOYSA-N	ketone	-0.26 ± 0.18 (8)	-0.38 ± 0.14 (9)	-0.41 ± 0.06 (8)	-0.52 ± 0.12 (8)	-0.01 ± 0.1 (6)
nonanone	VKCYHJWLYTUGCC-UHFFFAOYSA-N	ketone	0.18 ± 0.1 (11)	-0.32 ± 0.09 (8)	-0.15 ± 0.06 (11)	-0.2 ± 0.12 (12)	0.89 ± 0.12 (6)
acetyl furan	IEMMBWWQXVXBEU-UHFFFAOYSA-N	other	0.73 ± 0.18 (8)	NA	-0.73 ± 0.11 (8)	0.26 ± 0.2 (9)	0.23 ± 0.09 (6)
beta-butyrolactone	GSCLMSFRWBPUSK-UHFFFAOYSA-N	other	0.47 ± 0.17 (9)	1.21 ± 0.29 (9)	0.11 ± 0.09 (8)	1.04 ± 0.13 (8)	-0.18 ± 0.1 (6)
furfural	HYBBIBNJHNGZAN-UHFFFAOYSA-N	other	0.03 ± 0.15 (7)	-0.59 ± 0.25 (9)	-0.93 ± 0.2 (9)	1.12 ± 0.14 (8)	-0.14 ± 0.06 (6)
gamma-butyrolactone	YEJRWHAVMIAJKC-UHFFFAOYSA-N	other	NA	NA	0.65 ± 0.08 (15)	NA	NA
gamma-valerolactone	GAEKPEKOJKCEMS-UHFFFAOYSA-N	other	0.45 ± 0.16 (8)	-0.38 ± 0.12 (9)	-0.1 ± 0.06 (7)	0.61 ± 0.07 (9)	0.33 ± 0.1 (5)
oil	solvent	other	0 ± 0.02 (242)	0 ± 0.01 (122)	0 ± 0.01 (263)	0 ± 0.02 (268)	0 ± 0.02 (126)
γ-heptalactone	VLSVVMPLPMNWBH-UHFFFAOYSA-N	other	0.6 ± 0.09 (11)	-0.14 ± 0.08 (9)	-0.28 ± 0.06 (11)	1.01 ± 0.15 (13)	1.05 ± 0.14 (6)
1,8-cineole	WEEGYLXZBRQIMU-WAAGHKOSSA-N	terpene	0.32 ± 0.1 (10)	-0.21 ± 0.11 (7)	0.12 ± 0.07 (8)	2.73 ± 0.29 (9)	0.24 ± 0.05 (7)
(1R)-(-)-fenchone	LHXDLQBQYFFVNW-OIBJUYFYSA-N	terpene	0.47 ± 0.1 (10)	-0.26 ± 0.09 (8)	0.08 ± 0.06 (8)	3.23 ± 0.37 (9)	0.34 ± 0.06 (7)
(1R)-(-)-myrtenal	KMRMUZKLFIEVAO-UHFFFAOYSA-N	terpene	-0.1 ± 0.11 (10)	-0.29 ± 0.08 (8)	0.26 ± 0.09 (11)	-0.09 ± 0.16 (10)	0.2 ± 0.07 (7)
alpha-bisabolol	RGZSQWQPBWRIAQ-CABCVRRESA-N	terpene	-0.06 ± 0.09 (10)	-0.24 ± 0.11 (8)	-0.12 ± 0.34 (11)	0 ± 0.11 (12)	0.13 ± 0.04 (7)
alpha-ionone	UZFLPKAIBPNNCA-BQYQJAHWSA-N	terpene	-0.11 ± 0.07 (10)	-0.16 ± 0.08 (8)	0.21 ± 0.06 (11)	3.16 ± 0.39 (10)	0.33 ± 0.05 (8)
(+)-alpha-pinene	GRWFGVWFFZKLTI-RKDXNWHRSA-N	terpene	0.56 ± 0.13 (9)	-0.21 ± 0.1 (8)	0.07 ± 0.04 (6)	-0.36 ± 0.09 (9)	0.1 ± 0.08 (7)
alpha-terpineol	WUOACPNHFRMFPN-SECBINFHSA-N	terpene	-0.09 ± 0.12 (10)	-0.5 ± 0.14 (8)	0.19 ± 0.05 (11)	1.26 ± 0.19 (10)	1.75 ± 0.2 (8)
(-)-alpha-thujone	USMNOWBWPHYOEA-MRTMQBJTSA-N	terpene	0.12 ± 0.11 (11)	-0.23 ± 0.07 (8)	0.04 ± 0.07 (11)	-0.67 ± 0.18 (13)	0.47 ± 0.16 (8)
beta-citronellol	QMVPMAAFGQKVCJ-SNVBAGLBSA-N	terpene	-0.24 ± 0.07 (11)	-0.26 ± 0.11 (8)	NA	-0.07 ± 0.08 (12)	1.53 ± 0.1 (7)
beta-ionone	PSQYTAPXSHCGMF-BQYQJAHWSA-N	terpene	0.02 ± 0.15 (8)	-0.18 ± 0.1 (8)	0.18 ± 0.09 (8)	0.92 ± 0.18 (9)	0.21 ± 0.08 (8)
citral	WTEVQBCEXWBHNA-JXMROGBWSA-N	terpene	0.15 ± 0.17 (8)	-0.51 ± 0.16 (8)	0.19 ± 0.09 (6)	0.14 ± 0.17 (8)	0.84 ± 0.07 (7)
E,E-farnesol	CRDAMVZIKSXKFV-YFVJMOTDSA-N	terpene	0 ± 0.11 (10)	-0.55 ± 0.15 (8)	0.17 ± 0.06 (11)	0.17 ± 0.07 (11)	0.35 ± 0.04 (7)
geraniol	GLZPCOQZEFWAFX-JXMROGBWSA-N	terpene	-0.32 ± 0.05 (10)	-0.36 ± 0.08 (8)	0.05 ± 0.06 (11)	-0.38 ± 0.15 (10)	1.2 ± 0.08 (8)
geranyl acetate	HIGQPQRQIQDZMP-DHZHZOJOSA-N	terpene	-0.07 ± 0.1 (10)	NA	0.09 ± 0.09 (11)	0.15 ± 0.18 (8)	0.5 ± 0.19 (7)
linalool	CDOSHBSSFJOMGT-UHFFFAOYSA-N	terpene	-0.15 ± 0.06 (10)	-0.75 ± 0.14 (8)	0.02 ± 0.06 (10)	0.5 ± 0.18 (10)	1.6 ± 0.15 (8)
(R)-(-)-carvone	ULDHMXUKGWMISQ-SECBINFHSA-N	terpene	-0.06 ± 0.12 (10)	-0.4 ± 0.11 (8)	0.27 ± 0.06 (11)	-0.15 ± 0.11 (11)	1.38 ± 0.13 (7)
(R)-(+)-limonene	XMGQYMWWDOXHJM-JTQLQIEISA-N	terpene	0.3 ± 0.07 (10)	-0.15 ± 0.08 (8)	0.05 ± 0.04 (7)	-0.04 ± 0.14 (9)	0.22 ± 0.08 (7)
(R)-(+)-pulegon	NZGWDASTMWDZIW-MRVPVSSYSA-N	terpene	0.62 ± 0.14 (9)	-0.37 ± 0.12 (8)	0.33 ± 0.05 (8)	0.8 ± 0.23 (9)	0.63 ± 0.07 (7)
(S)-(+)-carvone	ULDHMXUKGWMISQ-VIFPVBQESA-N	terpene	0.13 ± 0.07 (10)	-0.36 ± 0.11 (8)	0.86 ± 0.02 (275)	-0.01 ± 0.1 (11)	1.3 ± 0.08 (7)
(S)-(-)-citronellal	NEHNMFOYXAPHSD-JTQLQIEISA-N	terpene	-0.13 ± 0.18 (9)	-0.36 ± 0.12 (8)	0.1 ± 0.11 (8)	1.65 ± 0.12 (8)	0.81 ± 0.03 (7)
(-)-trans-caryophyllene	NPNUFJAVOOONJE-GFUGXAQUSA-N	terpene	-0.07 ± 0.09 (10)	-0.25 ± 0.09 (8)	0.11 ± 0.08 (11)	1.58 ± 0.24 (12)	0.07 ± 0.07 (7)

References

1. Dweck, H. K. M. *et al.* Pheromones mediating copulation and attraction in drosophila. *PNAS* **112**, 2829–2835 (2015). DOI:10.1073/pnas.1504527112.
2. Stensmyr, M. C. *et al.* A conserved dedicated olfactory circuit for detecting harmful microbes in drosophila. *Cell* **151**, 1345–1357 (2012). DOI:10.1016/j.cell.2012.09.046.
3. de Bruyne, M., Clyne, P. J. & Carlson, J. R. Odor coding in a model olfactory organ: TheDrosophila maxillary palp. *J. Neurosci.* **19**, 4520–4532 (1999).
4. de Bruyne, M., Foster, K. & Carlson, J. R. Odor coding in the drosophila antenna. *Neuron* **30**, 537–552 (2001). DOI:10.1016/S0896-6273(01)00289-6.
5. Dobritsa, A. A., van der Goes van Naters, W., Warr, C. G., Steinbrecht, R. A. & Carlson, J. R. Integrating the molecular and cellular basis of odor coding in the drosophila antenna. *Neuron* **37**, 827–841 (2003). DOI:10.1016/S0896-6273(03)00094-1.
6. Stensmyr, M. C., Giordano, E., Balloi, A., Angioy, A.-M. & Hansson, B. S. Novel natural ligands for drosophila olfactory receptor neurones. *J. Exp. Biol.* **206**, 715–724 (2003). DOI:10.1242/jeb.00143.
7. Hallem, E. A., Ho, M. G. & Carlson, J. R. The molecular basis of odor coding in the drosophila antenna. *Cell* **117**, 965–979 (2004). DOI:10.1016/j.cell.2004.05.012.
8. Goldman, A. L., Van der Goes van Naters, W., Lessing, D., Warr, C. G. & Carlson, J. R. Coexpression of two functional odor receptors in one neuron. *Neuron* **45**, 661–666 (2005). DOI:10.1016/j.neuron.2005.01.025.
9. Kreher, S. A., Kwon, J. Y., Carlson, J. R. & Haven, N. The molecular basis of odor coding in the drosophila larva. *Neuron* **46**, 445–456 (2005). DOI:10.1016/j.neuron.2005.04.007.
10. Pelz, D. *Functional Characterization of Drosophila melanogaster Olfactory Receptor Neurons*. Doctoral thesis, Freie Universität Berlin, Berlin (2005).
11. Yao, C. A., Ignell, R. & Carlson, J. R. Chemosensory coding by neurons in the coeloconic sensilla of the drosophila antenna. *J. Neurosci.* **25**, 8359–8367 (2005). DOI:10.1523/JNEUROSCI.2432-05.2005.
12. Hallem, E. A. & Carlson, J. R. Coding of odors by a receptor repertoire. *Cell* **125**, 143–60 (2006). DOI:10.1016/j.cell.2006.01.050.
13. Pelz, D., Roeske, T., Syed, Z., de Bruyne, M. & Galizia, C. G. The molecular receptive range of an olfactory receptor in vivo (drosophila melanogaster or22a). *J. Neurobiol.* **66**, 1544–1563 (2006). DOI:10.1002/neu.20333.
14. Kwon, J. Y., Dahanukar, A., a Weiss, L. & Carlson, J. R. The molecular basis of CO2 reception in drosophila. *Proc. Natl. Acad. Sci. U. S. A.* **104**, 3574–3578 (2007). DOI:10.1073/pnas.0700079104.
15. Nissler, A. *Ligand search for genetically identified Drosophila Olfactory Receptors using Calcium-Imaging*. Bachelor's thesis, Universität Konstanz, Konstanz (2007).
16. Schmuker, M., de Bruyne, M., Hähnel, M. & Schneider, G. Predicting olfactory receptor neuron responses from odorant structure. *Chem. Cent. J.* **1** (2007). DOI:10.1186/1752-153X-1-11.
17. van der Goes van Naters, W. & Carlson, J. R. Receptors and neurons for fly odors in drosophila. *Curr. Biol. CB* **17**, 606–612 (2007). DOI:10.1016/j.cub.2007.02.043.
18. Kreher, S. A., Mathew, D., Kim, J. & Carlson, J. R. Translation of sensory input into behavioral output via an olfactory system. *Neuron* **59**, 110–124 (2008). DOI:10.1016/j.neuron.2008.06.010.
19. Turner, S. L. & Ray, A. Modification of CO2 avoidance behaviour in drosophila by inhibitory odorants. *Nature* **461**, 277–281 (2009). DOI:10.1038/nature08295.
20. Galizia, C. G., Münch, D., Strauch, M., Nissler, A. & Ma, S. Integrating heterogeneous odor response data into a common response model: A DoOR to the complete olfactome. *Chem. Senses* **35**, 551–563 (2010). DOI:10.1093/chemse/bjq042.
21. de Bruyne, M., Smart, R., Zammit, E. & Warr, C. G. Functional and molecular evolution of olfactory neurons and receptors for aliphatic esters across the drosophila genus. *J. Comp. Physiol. A* **196**, 97–109 (2010). DOI:10.1007/s00359-009-0496-6.
22. Marshall, B., Warr, C. G. & de Bruyne, M. Detection of volatile indicators of illicit substances by the olfactory receptors of drosophila melanogaster. *Chem. Senses* **35**, 613–625 (2010). DOI:10.1093/chemse/bjq050.
23. Montague, S. A., Mathew, D. & Carlson, J. R. Similar odorants elicit different behavioral and physiological responses, some supersustained. *J. Neurosci.* **31**, 7891–7899 (2011). DOI:10.1523/JNEUROSCI.6254-10.2011.

24. Silbering, A. F. *et al.* Complementary function and integrated wiring of the evolutionarily distinct drosophila olfactory subsystems. *J. Neurosci.* **31**, 13357–13375 (2011). DOI:10.1523/JNEUROSCI.2360-11.2011.

25. Dweck, H. K. M. *et al.* Olfactory preference for egg laying on citrus substrates in drosophila. *Curr. Biol.* **23**, 2472–2480 (2013). DOI:10.1016/j.cub.2013.10.047.

26. Gabler, S., Soelter, J., Hussain, T., Sachse, S. & Schmuker, M. Physicochemical vs. vibrational descriptors for prediction of odor receptor responses. *Mol. Inform.* **32**, 855–865 (2013). DOI:10.1002/minf.201300037.

27. Ronderos, D. S., Lin, C.-C., Potter, C. J. & Smith, D. P. Farnesol-detecting olfactory neurons in drosophila. *J. Neurosci.* **34**, 3959–3968 (2014). DOI:10.1523/JNEUROSCI.4582-13.2014.

28. Dweck, H. K. M., Ebrahim, S. A. M., Farhan, A., Hansson, B. S. & Stensmyr, M. C. Olfactory proxy detection of dietary antioxidants in drosophila. *Curr. Biol.* **25**, 455–466 (2015). DOI:10.1016/j.cub.2014.11.062.
